# The protective effect of Schisandrin C against methicillin-resistant *Staphylococcus aureus*-induced otitis media

**DOI:** 10.1128/aac.00095-26

**Published:** 2026-06-10

**Authors:** Weifang Sun, Meihui Tian, Xingye Wang, Shuang Jiang, Mengli Jin, Yan Wang, Yating Tang, Shuyue Zhu, Wenlu Liao, Xueying Ding, Xuanyu Lv, Huan Liu, Wu Song, Lin Wei, Yong Tang

**Affiliations:** 1 Traditional Chinese Medicine College, Changchun University of Chinese Medicine 159345, Changchun, Jilin, China; 2 Department of Traditional Chinese Medicine, the Fourth Affiliated Hospital of School of Medicine, and International School of Medicine, International Institutes of Medicine, Zhejiang University 593059 , Yiwu, Zhejiang, China; 3 School of Health Management, Changchun University of Chinese Medicine 159345, Changchun, China; 4 School of Basic Medical Science, Changchun University of Chinese Medicine 159345, Changchun, China; 5 College of Integrated Chinese and Western Medicine, Changchun University of Chinese Medicine 159345, Changchun, China; 6 School of Pharmaceutical Sciences, Changchun University of Chinese Medicine 159345, Changchun, China; 7 Department of Otorhinolaryngology–Head and Neck Surgery, The People’s Hospital of Jilin Province, Changchun, China; The Peter Doherty Institute for Infection and Immunity, Melbourne, Victoria, Australia

**Keywords:** Schisandrin C, MRSA, sortase A inhibitor, antivirulence, biofilm, otitis media

## Abstract

Methicillin-resistant *Staphylococcus aureus* (MRSA) is a significant pathogen in otitis media (OM), including acute otitis media (AOM) and chronic suppurative otitis media (CSOM), presenting considerable therapeutic challenges due to its biofilm formation, host immune evasion, and promotion of persistent inflammation. This study evaluated the therapeutic potential of Schisandrin C (Sch C), a natural lignan derived from *Schisandra chinensis*, in mitigating MRSA-induced OM. In rat models of MRSA-induced AOM and CSOM, Sch C significantly reduced bacterial load and inflammatory responses by decreasing pro-inflammatory cytokine levels. By upregulating tight junction protein ZO-1 and downregulating mucin MUC5B, it also restored epithelial barrier integrity. In addition, Sch C treatment improved auditory function. Mechanistic studies revealed that Sch C directly inhibited sortase A (SrtA), a key enzyme responsible for anchoring surface virulence factors in MRSA, by binding to its active site and inhibiting its transpeptidase activity (fluorescence resonance energy transfer IC₅₀ = 38.68 µM). Through inhibiting SrtA, Sch C disrupted MRSA adhesion, invasion, and biofilm formation without exerting direct bactericidal effects. Furthermore, Sch C synergized with ofloxacin, enhancing its efficacy against MRSA otitis by reducing bacterial load, inflammation, and tissue damage. Collectively, these findings establish Sch C as a potent *S. aureus* SrtA inhibitor, highlighting its promise as a therapeutic strategy for MRSA-induced OM.

## INTRODUCTION

Antimicrobial resistance has been recognized as one of the greatest public health threats of the 21st century, with the World Health Organization warning that resistant pathogens may cause up to 10 million deaths annually by 2050. Among these pathogens, methicillin-resistant *Staphylococcus aureus* (MRSA) stands out as a particularly formidable threat due to its multidrug resistance to β-lactam antibiotics and its ability to cause severe infections (1).

In the field of otology, MRSA has emerged as an increasingly important pathogen in otitis media (OM), particularly in recurrent or treatment-refractory cases (2). Among the different subtypes of OM, chronic suppurative otitis media (CSOM) represents the most severe and persistent form, defined by tympanic membrane (TM) perforation and continuous otorrhea, affecting over 330 million individuals worldwide (3). Beyond its high prevalence, CSOM contributes to substantial morbidity by causing conductive or sensorineural hearing loss and remains a major cause of life-threatening complications such as intracranial infections and mastoiditis (4). Despite antibiotics remaining the cornerstone of treatment, progress in developing novel therapeutic options for OM has been minimal over the past decade. Furthermore, repeated surgeries combined with inappropriate antibiotic use have exacerbated treatment failure and contributed to the growing problem of antimicrobial resistance (5, 6). These challenges underscore the urgent need for novel, non-antibiotic therapeutics; antivirulence strategies represent a promising approach to counter MRSA-induced OM and circumvent the limitations of existing therapies (7, 8). Unlike conventional antibiotics, antivirulence strategies target bacterial pathogenicity without exerting direct bactericidal pressure (9).

*S. aureus* produces a large number of virulence and immune evasion factors, which help the bacteria adhere to the mucosal surface of the host, thus hindering the human immune response (10). Among potential targets, the carotenoid biosynthetic pathway, the accessory gene regulator, and sortase enzymes have drawn considerable interest (11). Sortase A (SrtA), a membrane-bound transpeptidase first discovered by Mazmanian et al., plays a central role in *S. aureus* pathogenesis by anchoring surface proteins to the cell wall (12, 13). SrtA cleaves substrates at the conserved LPXTG motif, linking the newly exposed threonine to pentaglycine cross-bridges, thereby stabilizing proteins essential for adhesion and immune evasion (14). In *S. aureus*, approximately 20 surface proteins contain LPXTG signals, underscoring the importance of SrtA in infection (15). Mutants lacking SrtA show impaired protein anchoring and reduced virulence (16). Importantly, as SrtA is dispensable for bacterial survival *in vitro*, selective inhibition of SrtA is unlikely to impose strong selective pressure or foster antibiotic resistance, making it a promising target (11).

In addition to virulence factor-mediated pathogenicity, MRSA disrupts the host epithelial barrier, thereby intensifying inflammation and sustaining infection. It impairs mucosal defense mechanisms, leading to impaired clearance and increased bacterial colonization (17, 18). Clinical observations further suggest that such barrier dysfunction contributes to effusion formation, persistent inflammation, and hearing impairment (19). Collectively, these findings underscore the importance of therapeutic strategies that not only block bacterial adhesion but also restore epithelial barrier function, providing a promising avenue for the management of MRSA-induced OM.

To date, various classes of SrtA inhibitors have been reported, including natural products, synthetic small molecules, and peptide mimetics (20, 21). Among them, natural compounds are particularly attractive due to their structural diversity, pharmacological versatility, and favorable safety profiles. Schisandrin C (Sch C), a dibenzocyclooctadiene lignan extracted from *Schisandra chinensis*, is one of the most pharmacologically active components of this traditional Chinese medicinal herb. Previous studies have demonstrated that Sch C enhances host barrier function and mitigates inflammation, as evidenced by its ability to alleviate *Pseudomonas aeruginosa*-induced intestinal disruption in *Caenorhabditis elegans* and to improve ethanol-induced liver and intestinal injury via NF-κB and p38 MAPK pathway regulation. These pharmacological features suggest its potential value in infectious diseases. Despite lacking direct antibacterial activity, its anti-inflammatory and antioxidant effects provide a strong rationale for exploring its possible antivirulence role in *S. aureus* infections (22, 23). Furthermore, many natural polyphenols have recently been identified as potent antivirulence agents due to their ability to competitively bind to bacterial surface protein anchors (24, 25). Given its unique dibenzocyclooctadiene skeleton, we hypothesized that Sch C might also interfere with critical bacterial virulence factors.

Given its potential to both preserve host defense and attenuate bacterial pathogenicity, Sch C was investigated in this study as a potential SrtA inhibitor in MRSA-associated OM. Our initial molecular docking screening suggested a high binding affinity between Sch C and the SrtA enzyme. We systematically evaluated its ability to suppress SrtA-mediated bacterial adhesion, biofilm formation, and immune evasion, while simultaneously restoring epithelial barrier integrity via regulation of MUC5B and ZO-1. Moreover, we assessed the synergistic effects of Sch C with ofloxacin (OFL) in rat models of acute otitis media (AOM) and CSOM. The interactions between Sch C and SrtA were further characterized using molecular dynamics simulations and surface plasmon resonance (SPR). Collectively, our findings provide both mechanistic and translational evidence that Sch C attenuates MRSA virulence and preserves mucosal barrier function, establishing it as a promising therapeutic agent targeting SrtA in combination with conventional antibiotics for MRSA-induced OM.

## MATERIALS AND METHODS

### Bacterial strains, plasmids, culture conditions, and reagents

This investigation received endorsement from the Biosafety Committee of Changchun University of Chinese Medicine. *S. aureus* strains BAA-1717 (USA300) and Newman were procured from American Type Culture Collection (Manassas, VA, USA). The Δ*srtA* mutant of *S. aureus* USA300 and the pET-28a::SrtA plasmid were maintained in our laboratory. These strains were employed in the current research and cultured in brain heart infusion (BHI) broth (Hopebio, Qingdao, China) at 37°C. Both *S. aureus* and *Escherichia coli* were cultivated in tryptic soy broth (TSB, Hopebio, Qingdao, China) or Luria-Bertani (LB, Hopebio, Qingdao, China) broth. The fluorescent peptide Abz-LPATG-Dap(Dnp)-NH_2_ was produced by LifeTein LLC. Sch C (purity ≥99%) was obtained from Weikeqi Biotech Co., Ltd. (Chengdu, China, Fig. S1), while the antibiotics were obtained from Yuanye Biotech Co., Ltd. (Shanghai, China). OFL was purchased from Sigma-Aldrich (USA).

### Rat model of AOM

Male Sprague-Dawley (SD) rats, specifically of the specific pathogen-free grade and aged 6 to 8 weeks, were acquired from Liaoning Changsheng Biotechnology Co., Ltd. (Shenyang, Liaoning, China; license no. Sch CXK [Liao] 2020-0001). Rats were euthanized under deep isoflurane inhalation anesthesia. The rats were kept in a standard laboratory environment with unrestricted access to food and water.

A model of AOM was established by introducing *S. aureus* USA300 into the tympanic cavity via TM puncture (26, 27). Prior to the experiment, rats were acclimatized for 1 week (28). The integrity and normalcy of the external auditory canal, TM, and middle ear were confirmed through otoscopic examination, acoustic impedance measurements, and auditory brainstem response (ABR) testing.

USA300 was cultured in TSB at a dilution of 1:100 and incubated to the mid-logarithmic growth phase. The bacterial cells were harvested by centrifugation, washed three times with phosphate-buffered saline (PBS), and resuspended. To ensure a standardized inoculum, microbial suspensions with a defined optical density were prepared using the 1.0 McFarland turbidity standard and further adjusted to a final concentration of 1 × 10⁸ colony-forming units (CFU)/mL. Both external auditory canals were cleaned and disinfected using 75% ethanol. A total of 100 μL of the prepared *S. aureus* suspension (1 × 10⁸ CFU/mL) was administered into the bilateral tympanic cavities via TM puncture using a microsyringe to induce AOM.

**Fig 1:**
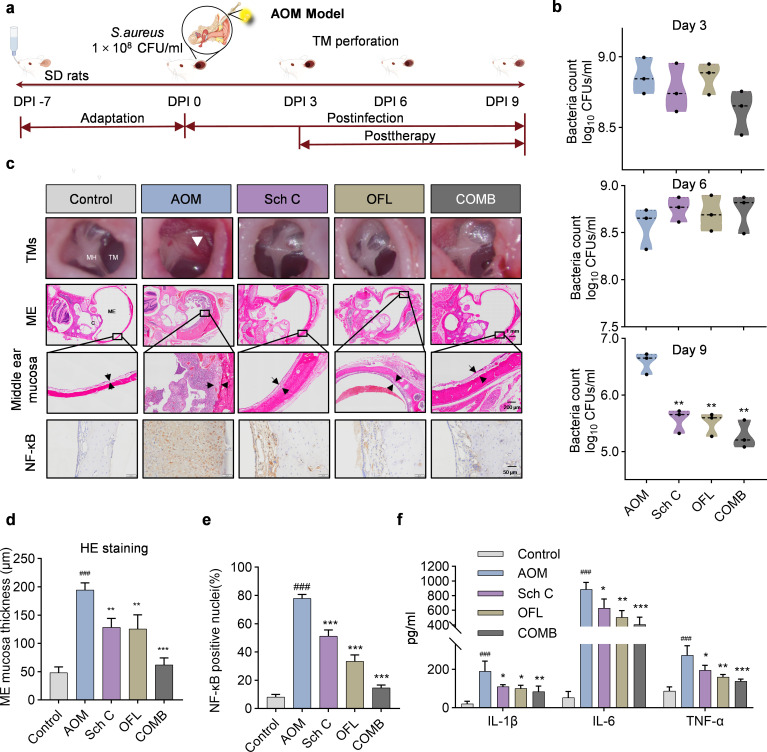
Combined administration of Sch and OFL significantly attenuates inflammation and accelerates mucosal repair in a rat model of AOM. (**a**) Schematic diagram of the experimental design for establishing an AOM rat model by TM puncture and intratympanic inoculation with MRSA (USA300) (created with Figdraw). (**b**) Bacterial load in middle ear lavage fluid on days 3, 6, and 9 post-inoculation in each group (*n* = 3). (**c**) Therapeutic effects of Sch C, OFL, and COMB on TM recovery in AOM rats, the histopathology of the middle ear detected by hematoxylin and eosin (H&E) staining, and representative middle ear tissue immunohistochemical staining of NF-κB (scale bar: 200 μm). (**d**) Quantitative analysis of middle ear mucosal thickness (*n* = 3). (**e**) Statistical analysis of NF-κB inflammatory marker expression in middle ear tissues. (**f**) Enzyme-linked immunosorbent assay (ELISA) results showing the levels of pro-inflammatory cytokines IL-1β, IL-6, and TNF-α in middle ear lavage fluid across different treatment groups (*n* = 3). Data are presented as mean ± SD and analyzed using one-way analysis of variance (ANOVA): * indicates *P* < 0.05, ** indicates *P* < 0.01, and *** indicates *P* < 0.001 vs the AOM group; ### indicates *P* < 0.001 vs control group.

The SD rats were randomly divided into five distinct groups: control group (PBS group), AOM group (USA300 + PBS), Sch C group (USA300 + Sch C 2.60 mM·d⁻¹), OFL group (USA300 + 0.3% OFL 8.30 mM·d⁻¹), and the COMB group (USA300 + Sch C 1 2.60 mM·d⁻¹ + 0.3% OFL 8.30 mM·d⁻¹), with 12 rats allocated to each group. Treatment commenced on DPI (day post-infection) 3 post-infection and continued through DPI 9. The day of initial MRSA inoculation was defined as DPI 0. Following the procedure described in the model establishment section, 20 µL of each drug formulation was carefully instilled into the middle ear cavity using a microsyringe. After administration, rats were maintained in a ventral recumbent position for approximately 5 min to facilitate even distribution of the solution, after which 2% isoflurane anesthesia was discontinued and the animals were allowed to recover spontaneously. Middle ear lavage fluid was collected from three randomly selected rats in each group on DPI 3, 6, and 9. On DPI 9 after modeling, the remaining three rats in each group were anesthetized by intraperitoneal injection of 10 g/L pentobarbital sodium at a dosage of 30 mL/kg for general anesthesia, followed by tympanic membrane imaging and auditory bulla collection for subsequent analyses (Fig. 1a).

### Rat model of CSOM

**Fig 2:**
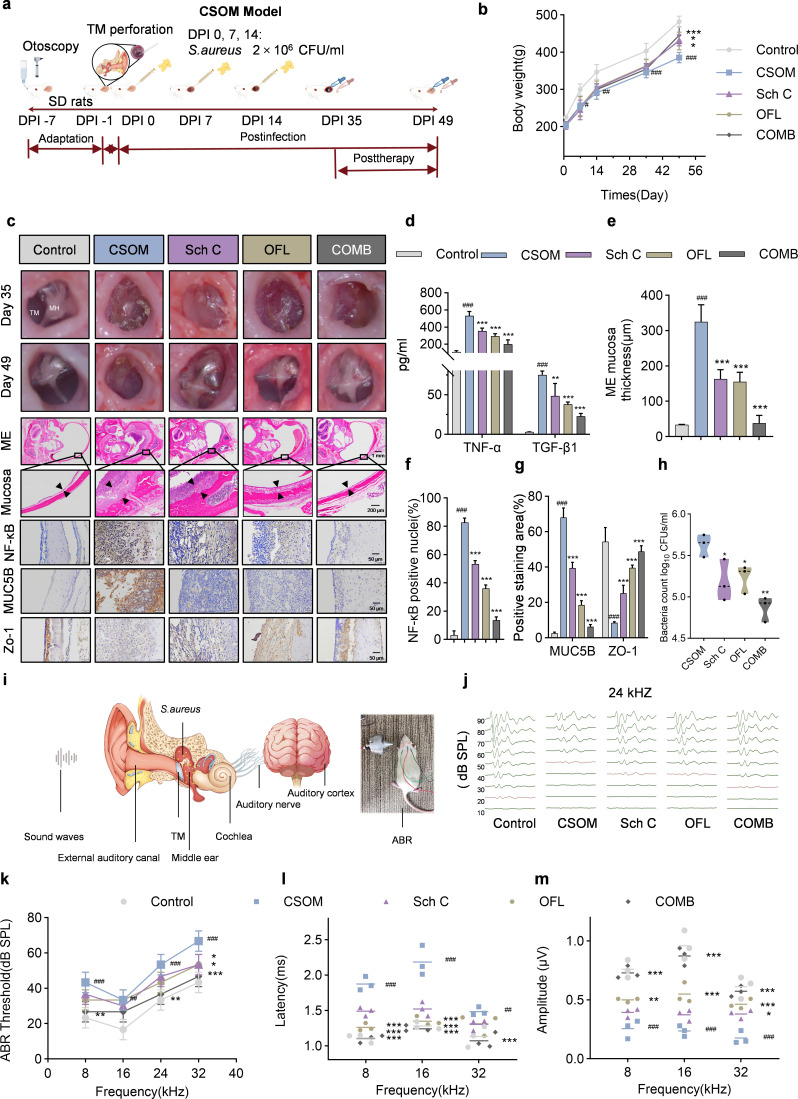
Effects of Sch C and OFL on inflammation and mucosal changes in rats with CSOM. (**a**) Schematic illustration of the CSOM experimental design (created with Figdraw). (**b**) Body weight changes in each group on days 1, 7, 14, 35, and 49 post-induction (*n* = 3). (**c**) Representative TM images from each group on day 35 and 49 showing morphological changes. Therapeutic effects of Sch C, OFL, and COMB on TM recovery in AOM rats, as assessed by H&E staining of middle ear histopathology and immunohistochemical staining of NF-κB in middle ear tissues (scale bar: 200 μm). (**d**) ELISA analysis of TNF-α and TGF-β1 levels in middle ear lavage fluid from each group (*n* = 3). (**e**) The quantitative analysis of middle ear mucosal thickness (*n* = 3). (**f, g**) Quantitative immunohistochemical analysis of inflammatory markers in middle ear tissues (scale bar: 200 μm). (**h**) Quantification of bacterial load in middle ear lavage fluid at DPI 49 across different experimental groups in the CSOM study (*n* = 3). (**i**) Schematic diagram of sound conduction, middle ear anatomy, and a photograph of the ABR experimental setup (created with Figdraw). (**j**) Typical ABR waveforms at a frequency of 24 kHz in each group. (**k–m**) ABR thresholds, latencies, and wave I amplitudes measured at different stimulus frequencies on day 49. Data are shown as mean ± SD and were analyzed using one-way ANOVA: * indicates *P* < 0.05, ** indicates *P* < 0.01, *** indicates *P* < 0.001 vs CSOM group, ### indicates *P* < 0.001 vs control group.

In order to investigate the impact of Sch C on the CSOM model in SD rats, we ensured that animal selection, pre-experimental examinations, acclimatization, and anesthesia procedures were consistent with the AOM protocol. Rats were euthanized as described for the AOM model under deep anesthesia. The establishment of the CSOM model followed a standardized timeline (Fig. 2a). A 100 µL inoculation of *S. aureus* (USA300) at a concentration of 2 × 10^6^ CFU/mL was administered into the middle ear through the bilateral TM under sterile conditions. The first inoculation was designated as DPI 0. To induce chronic infection, repeated inoculations were administered into the middle ear via the bilateral TM on DPI 0, 7, and 14. Body weight measurements were taken prior to each inoculation, and all injections were performed between 9 and 10 a.m. Treatment was initiated 3 weeks after the third inoculation (starting on DPI 35) and continued for 14 consecutive days. Following the treatment period, ABR testing was conducted on rats in each group.

The study comprised five groups: control group (PBS group), CSOM group (USA300), Sch C group (USA300 + Sch C 2.60 mM·d⁻¹·d⁻¹), OFL group (USA300 + 0.3% OFL 8.30 mM·d⁻¹), and the COMB group (USA300 + Sch C 2.60 mM·d⁻¹·d⁻¹ + 0.3% OFL 8.30 mM·d⁻¹), with 12 rats allocated to each group. On DPI 35 and 49, tympanic membrane imaging was performed on three randomly selected rats from each group. On DPI 49, after general anesthesia was induced by intraperitoneal injection of 10 g/L sodium pentobarbital at a dose of 30 mL/kg, three rats from each group were randomly selected for middle ear lavage fluid collection, and another three were used for hearing assessment and auditory bulla collection for subsequent analyses. The drug administration method was the same as that used for the AOM model.

### Bacterial load in a rat model of AOM

Bacterial load was assessed by lavaging the ears of three rats per group on DPI 3, 6, and 9 with 100 μL sterile saline. Then, 10 μL of a 1:100 dilution was plated on BHI agar, and CFU were counted after overnight incubation.

### ELISA

Levels of IL-1β, IL-6, TNF-α, and TGF-β1 in rat middle ear lavage fluid were determined using ELISA following the manufacturer’s instructions (MultiSciences Biotech, Hangzhou, China).

### H&E staining and immunohistochemistry

Middle ear cavities were collected, fixed in 4% paraformaldehyde for 24 h, decalcified with 10% EDTA for 4 weeks, and embedded in paraffin. Sections (4 μm) were stained with H&E (Beyotime) for morphological assessment. For IHC, sections were deparaffinized, rehydrated, and subjected to antigen retrieval in citrate buffer (pH 6.0). Slides were blocked with peroxidase solution and normal serum, then incubated overnight at 4°C with primary antibodies against MUC5B (1:400), NF-κB p65 (1:50), and ZO-1 (1:50). All IHC experiments were performed using the UltraSensitive SP (Mouse/Rabbit) IHC Kit (Fuzhou Maxin, China), which provides a standardized secondary antibody and DAB chromogenic system. Images were captured using an Olympus APX100 microscope; the positive staining area was quantified with ImageJ.

### ABR testing

Rats were anesthetized and placed on a thermal blanket. ABR thresholds were recorded using Tucker-Davis Technologies RZ6 with 1,024 stimulus repetitions. Electrodes were positioned subcutaneously (recording: midline between auricles; reference: behind test ear; ground: behind the contralateral ear). Tone bursts (8, 16, 24, and 32 kHz; 4 ms; 1 ms rise/fall) were presented from 90 dB SPL, decreasing in 10 dB steps until waveforms were undetectable, then increased by 5 dB.

### Cytotoxicity assay, live/dead, and LDH assays

**Fig 3:**
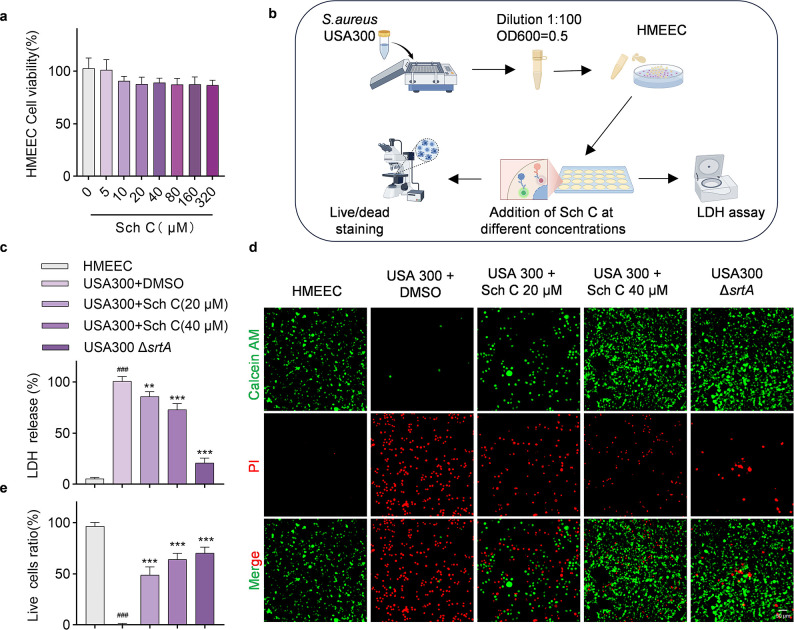
Sch C protects HMEECs from *S. aureus* USA300-mediated cytotoxicity. (**a**) The effect of different concentrations of Sch C on HMEECs viability was measured using the CCK-8 assay. (**b**) Workflow of live/dead staining and LDH assay in HMEECs infected with USA300 (created with Figdraw). (**c**) LDH release from HMEECs treated with different concentrations of Sch C. (**d, e**) Fluorescence imaging of live/dead cells stained with Calcein-AM (green for live cells) and propidium iodide (PI, red for dead cells) to assess cell survival. Scale bar: 100 μm. Data are presented as mean ± SD and analyzed using one-way ANOVA: * indicates *P* < 0.05, ** indicates *P* < 0.01, *** indicates *P* < 0.001 vs dimethyl sulfoxide (DMSO) group, ### indicates *P* < 0.001 vs control group. All experiments were performed in triplicate.

Human middle ear epithelial cells (HMEECs) were purchased from Shanghai Hexu Biotechnology Co., Ltd. Cells were cultured in a 1:1 mixture of bronchial epithelial basal medium and BEGM SingleQuots (Lonza Bioscience), supplemented with 10% fetal bovine serum and 1% penicillin/streptomycin (Gibco, Grand Island, NY, USA) (29). Cells were kept in a humidified incubator at 37°C with an atmosphere of 5% CO₂ and 95% humidity. HMEECs (1 × 10^4^ per well) were cultured in 96-well plates for 24 h, treated with varying Sch C concentrations (0, 5, 10, 20, 40, 80, 160, and 320 μM) for another 24 h, and assessed with CCK-8 at 450 nm. For infection assays, HMEECs were incubated with *S. aureus* USA300 in serum-free culture medium with Sch C for 5 h. Live/dead cells were imaged using a Calcein AM/PI kit (US EVERBRIGHT, Suzhou, China), and LDH release (Beyotime, Shanghai, China) was measured per manufacturer instructions (Fig. 3b).

### Expression and purification of recombinant SrtA protein

We employed the heat shock method to introduce both the original and mutant forms of the pET-28a::SrtA plasmid into the *Escherichia coli* BL21 (DE3) strain. *E. coli* BL21 (DE3) was inoculated into LB liquid medium containing kanamycin (50 mg/mL) at a 1:100 dilution and incubated overnight at 37°C with shaking at 200 r/min. Subsequently, the culture was re-inoculated at a 1:100 dilution and incubated in a shaker at 37°C for 4 h, after which isopropyl-β-D-thiogalactopyranoside (Sangon Biotech, Shanghai, China) was added to a final concentration of 0.5 mM. The induction for protein expression was carried out at 16°C for 6 h. Following induction, the cells were harvested by high-speed centrifugation at 10,000 r/min for 10 min. The resulting cell pellet was resuspended in binding buffer (50 mM Tris-HCl, 150 mM NaCl, 5 mM CaCl_2_), and then the bacterial cells underwent ultrasonication to induce cellular disruption. Proteins were purified via Ni-NTA affinity chromatography and analyzed by SDS-PAGE. Figure S3 displays grayscale SDS-PAGE images of the purified SrtA proteins.

### Bacterial growth, MIC, and disk diffusion

Overnight *S. aureus* cultures were diluted 1:100 in fresh medium. Growth curves were measured at an optical density at 600 nm (OD600) over 24 h with or without Sch C. MIC was determined via broth dilution (0 μM–320 μM Sch C, 0 μM–88 μM OFL). Disk diffusion was performed on TSA plates with bacterial lawns and sample-containing discs, and inhibition zones were measured after 24 h.

### Molecular docking and molecular dynamics simulation

The structure of SrtA was obtained from the Protein Data Bank (ID: 1T2P), and the three-dimensional structure of Sch C was constructed using the ChemBio3D Ultra 12.0 software package. Subsequently, molecular docking simulations of the protein-ligand interactions were performed using the AutoDock Vina 1.1.2 software. Molecular visualization was conducted using PyMOL (v.2.5.7) to reveal the details of the ligand-receptor interactions. Molecular dynamics simulations were performed in Gromacs 2022.3 for 100 ns; RMSD, RMSF, H-bonds, and Gibbs free energy were analyzed with GraphPad Prism.

### Surface plasmon resonance

Sch C was diluted in the analyte buffer to prepare six concentrations: 50, 25, 12.5, 6.25, 3.125, and 0 μM. Sch C was injected into channels Fc3 and Fc4 at a flow rate of 30 μL/min for an association phase lasting 120 s, followed by a 200 s dissociation phase. Both the association and dissociation phases were conducted in analyte buffer. Six cycles were performed in ascending order of analyte concentrations. After each interaction cycle, the analyte was allowed to dissociate naturally, and the chip did not require regeneration.

### Western blot

Different concentrations of Sch C were added to *S. aureus* USA300, followed by overnight incubation until the OD600 reached 2.0. The bacterial culture was centrifuged at 5,000 rpm for 5 min at 4°C, and the supernatant was collected. The total protein of *S. aureus* was extracted using the kit protocol and subsequently subjected to 12% SDS-PAGE electrophoresis, semi-dry transfer, and blocking with 2.5% skim milk. The membrane was washed with TBS-Tween and then incubated with rabbit anti-SrtA antibody (diluted 1:3,000) for 2 h on a shaking platform. After incubation with the primary antibody, the membrane was washed and incubated with a 1:5,000 dilution of horseradish peroxidase-labeled goat anti-rabbit secondary antibody at room temperature for 2 h. Finally, ECL substrate was added, and the membrane was exposed and imaged using a BeyoECL Plus system (Beyotime Biotechnology, Shanghai, China). Band intensity was quantified using ImageJ software (NIH, MD, USA). Prestained Protein Marker IV (10 kDa–200 kDa; cat. no. G2083-250UL, Servicebio, China) was used as the molecular weight marker in this study.

### SrtA activity assay (fluorescence resonance energy transfer [FRET])

SrtA is capable of recognizing and cleaving the Abz-LPATG-Dap(Dnp)-NH_2_ motif, leading to alterations in the fluorescence signal. We employed FRET to evaluate the activity of the SrtA transpeptidase. The experiment was performed in a 96-well black plate, where 100 μL of a mixture containing SrtA protein at a concentration of 10 μM and varying concentrations of Sch C was incubated for 1 h in a dark environment at 37°C. Subsequently, 10 μM of the fluorescent substrate peptide Abz-LPATG-Dap(Dnp)-NH_2_ was added, and the mixture was incubated at 37°C in the dark for an additional 20 min and Ex 309 nm/Em 420 nm.

### Biofilm formation, biofilm disruption, and determination of extracellular polysaccharides

Biofilm formation and disruption were examined using a modified version of a previously reported assay (30). Lyophilized rabbit plasma (100 μL, 20%, HopeBio, Qingdao, China) was added to each well of the microporous plate and coated overnight at 4°C. Subsequently, overnight cultures of *S. aureus* USA300 and Δ*srtA* were introduced into BHI medium containing 3% NaCl and 0.5% glucose at a dilution ratio of 1:100, and the bacteria were grown until the OD600 reached 0.3. Various concentrations of Sch C were mixed with the bacterial suspension and incubated at 37°C for 16 to 24 h to facilitate biofilm formation. After incubation, the supernatant was removed, and the plate was washed with PBS. The resulting biofilm was stained with crystal violet for 20 min, after which excess dye was washed away with PBS. Absolute ethanol was added to elute the bound dye, and the solution was transferred to a new microporous plate. The absorbance of the plates was measured at OD595 using a microplate reader. For biofilm disruption, Sch C was added after 24 h biofilm maturation.

For the biofilm checkerboard assay, Sch C and OFL were added alone or in combination to the bacterial suspension before biofilm formation in a two-dimensional checkerboard format using serial dilutions. The plates were then incubated under biofilm-forming conditions at 37°C. After incubation, the wells were washed, stained with crystal violet, and measured at OD595 as described above. Biofilm inhibition was calculated according to the following equation: Inhibition (%) = [1-$\frac{OD595\;of\;treated\;well}{OD595\;of\;untreated\;control\;well}$] × 100 (31).

Untreated biofilms receiving solvent alone were used as the control. The MBIC_90_ was defined as the lowest concentration of antibiotic/inhibitor that inhibited biofilm formation by at least 90% relative to the untreated control. Based on the MBIC_90_ values obtained for single-agent and combination treatments, the fractional biofilm inhibitory concentration (FBIC) was calculated as follows: FBIC = FBIC_A+_ FBIC_B_ = $\frac{MBIC90A\left(B\right)}{MBIC90A}$ + $\frac{MBIC90B\left(A\right)}{MBIC90B}$, where A represents the antibiotic (OFL) and B represents the inhibitor (Sch C); MBIC_90A(B)_ is the MBIC_90_ of OFL in the presence of Sch C, and MBIC_90 B(A)_ is the MBIC_90_ of Sch C in the presence of OFL. Drug interaction was interpreted as follows: FBIC ≤ 0.5, synergism; 0.5 < FBIC ≤ 1, additivity; 1 < FBIC < 4, indifference; and FBIC ≥ 4, antagonism (32).

The extracellular polysaccharides content was quantified using the phenol-sulfuric acid method (30, 33, 34).

### CLSM analysis of MRSA biofilm structure

A modified CLSM analysis was employed to examine the MRSA biofilm structure, based on previously described methods (35). The established biofilms were subjected to fluorescent staining using SYTO9 green fluorescent nucleic acid stain (Thermo Fisher Scientific, USA) for specific visualization of metabolically active bacterial cells. The stained specimens were then examined using a laser scanning confocal microscope (CLSM, FV3000, Olympus, Japan) to acquire high-resolution three-dimensional morphological data, which were finally rendered into 3D mode by Imaris software (Imaris, Bitplane AG).

### Fibronectin-binding assay

The fibronectin-binding assay was performed as previously described, with minor modifications (36). Ninety-six-well plates coated with 20 μg/mL fibrinogen, blocked with 5% BSA, washed, and incubated with *S. aureus* ± Sch C at 37°C for 2 h, then fixed with 4% formaldehyde and stained with crystal violet. Then, 100 μL of the eluted solution was transferred to a new 96-well plate and measured the absorbance at OD595 nm. The relative adhesion rate was calculated as (OD595 of the drug-treated group / OD595 of the control group) × 100%.

### FITC-IgG binding to SpA

The analysis of FITC-IgG binding to SpA was performed using a previously described method (37). *S. aureus* USA300 cultured in TSB ± Sch C to an OD600 of 1.0. Cells were fixed and stained with FITC-labeled rabbit anti-goat IgG (Proteintech, Wuhan, China) at 37°C for 2 h. After two PBS washes, the bacteria were resuspended in sterile PBS. Flow cytometry (Beckman Coulter, USA) was used to measure IgG binding, analyzing fluorescence intensity from 10,000 bacteria and calculating the median value.

### Colony spreading assay

Colony spreading assay was completed as described but with slight revision (38–40). *S. aureus* USA300 was cultured overnight in TSB medium and adjusted to an optical density (OD600) of 1. Subsequently, 10 μL of the overnight culture was inoculated onto the center of TSB plates containing 0.3% (wt/vol) soft agar and varying concentrations of Sch C. To ensure consistent surface moisture, a critical factor for spreading, the plates were sealed and incubated at 37°C for 16 h. Following incubation, photographs were taken and the area of the bacterial halo was measured.

### Statistical analysis

All experiments were performed at least three times. Data analysis was conducted using GraphPad Prism version 8.0. Results are presented as the mean ± standard deviation. One-way ANOVA was used to assess differences among groups, with significance levels indicated in the figure legends. *P* < 0.05 was considered statistically significant.

## RESULTS

### Establishment of a rat model of MRSA-induced AOM

We developed a rat model of AOM by injecting MRSA into the middle ear via the TM to evaluate the efficacy of Sch C against MRSA infection (Fig. 1a). Under the surgical microscope, the normal anatomical structures of the rat middle ear were clearly visualized, revealing that the external view of the TM and bulla exhibited intact, translucent structures without effusion (Fig. S2A). The middle ear mucosa appeared thin, smooth, and non-hyperemic, without visible exudates (Fig. S2B). The ossicular chain, comprising the malleus, incus, and stapes, was intact and well-formed (Fig. S2C). Within 1–3 days post-induction, a characteristic milky or turbid purulent effusion was observed in the tympanic cavity (black arrow), accompanied by congestion and mucosal thickening, indicating a successful acute inflammatory response (Fig. S2D).

### Protection of middle ear structures and modulation of inflammation in AOM by Sch C

To examine the protective impact of Sch C on AOM in rats infected with MRSA, lavages of the middle ears of rats were performed under sterile conditions. The CFU counts indicated that there were no statistically significant differences among the groups on days 3 and 6, compared with the AOM group. However, on day 9 post-treatment, the Sch C group showed an approximate 90.25% reduction in the bacterial CFU count of the middle ear lavage fluid (*P* < 0.01) (Fig. 1b).

Subsequently, the morphology of TM, histological changes, and immunohistochemical markers were evaluated to characterize the damage induced by MRSA and the protective effects of Sch C (Fig. 1c and d). The TM morphology was examined across different treatment groups using microscopy on day 9. Compared with the control group, the TM of the AOM group exhibited slight turbidity and hyperemia (a white triangular marker). Notably, the TM of rats in the Sch C, OFL, and COMB groups showed no signs of ear effusion, invagination, or pus discharge (Fig. 1c). H&E staining revealed that, relative to the control group, the mucosal fibrous layer in the AOM group exhibited thickening, infiltration of superficial inflammatory cells, the presence of new bone on the middle ear bone wall, and an increase in the thickness of the middle ear mucosa. Statistical analysis showed that treatment with Sch C, OFL, and COMB all led to a significant reduction in mucosal thickness compared with the AOM group. Specifically, while Sch C and OFL reduced thickness by approximately 33.80% and 35.33%, respectively (*P* < 0.01), the COMB group exhibited the most potent therapeutic effect, reducing thickness by 68.10% (*P* < 0.001), effectively restoring the mucosa to near-normal levels (Fig. 1d). Similarly, immunohistochemical staining demonstrated that the overexpression of NF-κB in the AOM group was significantly attenuated by all treatment interventions. Notably, the COMB group showed a superior inhibitory effect on NF-κB expression compared with either single-drug group (*P* < 0.001), suggesting a powerful anti-inflammatory synergy (Fig. 1e).

Furthermore, ELISA was employed to quantify the levels of IL-1β, IL-6, and TNF-α in the middle ear lavage fluid of infected rats. The results indicated that, in comparison to the AOM group, the levels of IL-1β, IL-6, and TNF-α were reduced to varying extents in the COMB, Sch C, and OFL groups (Fig. 1f). Collectively, these findings support the conclusion that Sch C effectively mitigates AOM resulting from MRSA infection.

### Establishment and characterization of a CSOM model

To establish a CSOM model, repeated trans-tympanic injections of *S. aureus* were administered into SD rats (Fig. 2a). The baseline conditions of the rats were observed and analyzed. The control group exhibited good mental states, normal weights, no ear canal secretions, normal ear appearances, and no abnormal behaviors such as head shaking or ear scratching, while maintaining a normal diet. In contrast, the CSOM group developed recurrent purulent ear secretions, displayed abnormal behaviors including frequent head shaking and ear scratching, and exhibited lower weights compared to the control group. The weights of rats in each group were recorded on days 0, 7, 14, 35, and 49. During the modeling period from day 0 to day 14, the weights of rats in each group increased. During the intervention period from days 35 to 49, the body weight gain in the Sch C group showed a trend similar to that of the control group (Fig. 2b). Additionally, H&E staining revealed evident local capillary dilation and congestion, signifying a pronounced chronic inflammatory response in the middle ear tissue (Fig. S2E). Together, these findings confirmed the successful establishment of the CSOM model and provided a foundation for subsequent therapeutic investigations.

### Sch C attenuated middle ear pathology and modulates inflammation in CSOM

We further assessed the pathological alterations in the TM and middle ear through histological analysis. As illustrated in Fig. 2c, microscopic observations indicated that the control group exhibited an intact, translucent TM with clear visibility of the malleus handle. On day 35, compared with the control group, the TMs of rats in the other groups exhibited pathological changes characterized by opacity, thickening, loss of normal luster, and congestion. The CSOM group showed more severe pathological alterations, characterized by pronounced opacity, marked thickening, loss of translucency, and intense vascular congestion, with evident yellowish deposits in the superior region on day 49. Treatment with Sch C partially restored TM clarity and structure, resulting in reduced thickening and improved translucency. Notably, the COMB group showed near-complete restoration of TM integrity and appearance, resembling the control group, suggesting a synergistic protective effect (Fig. 2c).

The H&E results demonstrated that the degree of inflammatory cell infiltration in the middle ear cavity of the three treatment groups was less severe compared to the CSOM group (Fig. 2c). The middle ear mucosa in the CSOM group exhibited significant epithelial thickening, architectural disorganization, and extensive infiltration of inflammatory cells in the subepithelial stroma, accompanied by partial glandular destruction. In addition, the thickness of the middle ear mucosa in the CSOM group was significantly greater, with increased inflammatory cell deposits. Quantitative analysis showed that the middle ear mucosal thickness reached 324.30 ± 48.32 µm in the CSOM group. While Sch C and OFL reduced this thickness by approximately 50% (*P* < 0.001), the COMB treatment resulted in a dramatic reduction to 38.10 ± 22.08 µm, representing an 88.25% decrease and nearly normalizing the mucosal morphology (*P* < 0.001, Fig. 2c and e). Furthermore, while Sch C treatment significantly decreased TNF-α and TGF-β1 levels in middle ear lavage fluid, the COMB group achieved the most pronounced suppression of these cytokines compared to either single-treatment group (*P* < 0.001, Fig. 2d). This superior outcome indicates that the combination of Sch C and OFL effectively alleviates the inflammatory response and epithelial barrier dysfunction.

Moreover, immunohistochemical staining revealed distinct molecular alterations in the middle ear tissues of rats with CSOM. Specifically, MUC5B, a major secretory mucin implicated in mucus hypersecretion, and NF-κB, a key transcription factor involved in inflammatory signaling, were markedly overexpressed in the CSOM group. Treatment with Sch C effectively reversed these pathological overexpressions (Fig. 2c and f). Additionally, compared to the CSOM group, all three treatment groups significantly upregulated the expression of ZO-1, an essential tight junction protein that maintains epithelial barrier integrity (Fig. 2c and g). At DPI 49, Sch C and OFL significantly reduced the bacterial load by approximately 61.04% and 59.07%, respectively, while the COMB group achieved a superior synergistic reduction of 82.50% compared to the CSOM group (Fig. 2h). Together, these findings demonstrate that Sch C alleviated TM and mucosal pathological damage, suppressed inflammatory responses, and restored epithelial barrier function in CSOM.

### ABR in CSOM with Sch C

In the context of OM, particularly CSOM, persistent inflammation and structural damage of the middle ear impair sound conduction and lead to elevated ABR thresholds. Moreover, prolonged inflammation may induce secondary damage to the inner ear and auditory neural pathways, further exacerbating hearing loss (Fig. 2i). Therefore, changes in ABR thresholds were assessed on day 35 post-treatment. The results indicated that, compared with the CSOM group, the Sch C group exhibited a significantly decreased ABR threshold at 32 kHz (*P* < 0.05), indicating that the Sch C group has a protective effect against hearing loss in rats with *S. aureus*-induced CSOM (Fig. 2j and k). In addition to ABR thresholds, latency and amplitude were also evaluated. Latency reflects the conduction velocity of the auditory pathway, with prolonged latency indicating delayed neural transmission. Amplitude represents the extent of neural synchrony, where reduced amplitude suggests impaired cochlear or neural function. On day 49, compared with the CSOM group, all treatment groups exhibited shorter wave I latencies and higher amplitudes, indicating further improvement in auditory function (Fig. 2l and m). Collectively, the results indicated that Sch C not only effectively alleviated the pathological changes associated with CSOM caused by MRSA infection but also further enhanced auditory function.

### Sch C inhibited the invasion of *S. aureus* into HMEECs

To investigate whether Sch C exhibited a protective effect against cellular damage, the cytotoxicity of Sch C in HMEECs was initially assessed using the CCK8 assay. The results indicated that Sch C under the 320 μM concentration did not significantly affect the viability of HMEECs (*P* > 0.05) (Fig. 3a). Next, we conducted Calcein/PI staining and LDH assays in HMEECs infected with USA300 to further examine cell injury (Fig. 3b). Treatment with Sch C significantly reduced LDH levels in the culture supernatant compared to the DMSO group, regardless of dose, reflecting reduced cell death (Fig. 3c).

To further explore the potential protective effect of Sch C on HMEECs infected with *S. aureus*, cell viability was evaluated using the Calcein/PI cell viability and cytotoxicity assay kit. Sch C (40 μM) treatment markedly protected HMEECs from *S. aureus*-induced cytotoxicity and substantially increased cell viability (Fig. 3d and e). Together, these findings demonstrate that Sch C effectively protects HMEECs from S. aureus-induced cytotoxicity, exhibiting a protective profile consistent with that observed in the SrtA-deficient strain.

### Sch C did not affect the growth of *S. aureus* USA300 and Newman

**Fig 4:**
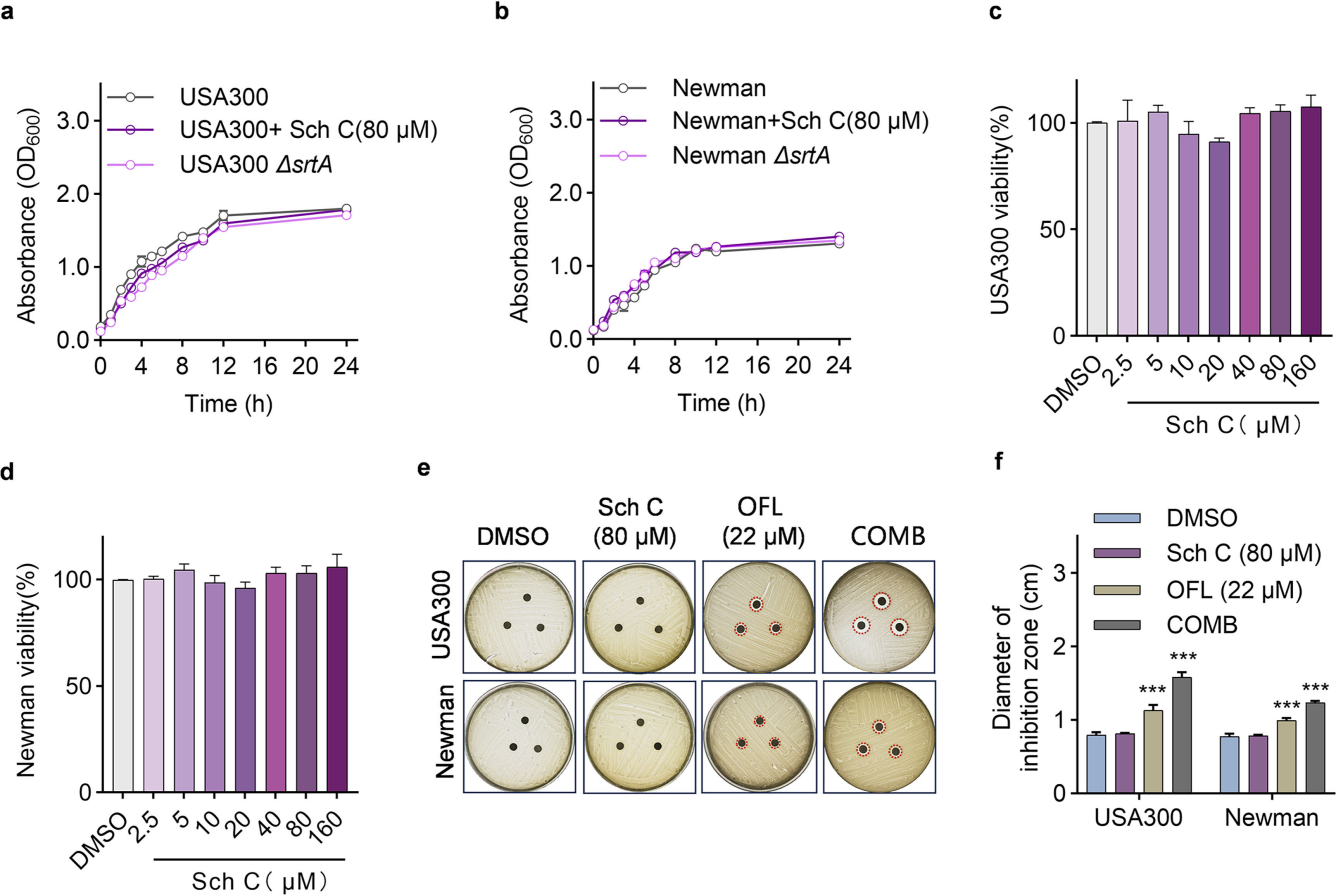
Sch C shows no growth inhibition or cytotoxicity against *S. aureus*. (**a, b**) Growth curves of *S. aureus* USA300 and Newman under three conditions: no treatment, treatment with Sch C (80 μM), and Δ*srtA* strain. (**c, d**) CCK-8 of *S. aureus* USA300 and Newman exposed to increasing concentrations of Sch C (2.5, 5, 10, 20, 40, 80, and 160 μM). (**e, f**) Zone of inhibition assay using the disc diffusion method to assess the antibacterial effects of Sch C and OFL against USA300 and Newman. Statistical significance was analyzed by one-way ANOVA: *** indicates *P* < 0.001 vs DMSO group. All experiments were performed in triplicate.

The MIC of Sch C against the USA300 and Newman strains was determined to exceed 320 μM, indicating a lack of inhibitory effect on *S. aureus* at these elevated concentrations. To investigate the effect of Sch C on the growth of *S. aureus* USA300 and Newman, we recorded the impact of 80 μM Sch C on both strains over a 24 h period. The growth curve indicated no observable effect on bacterial growth at any of the time points (Fig. 4a and b). Subsequently, we employed the CCK-8 method to assess the cytotoxic effects of Sch C on *S. aureus* USA300 and Newman. The results showed that Sch C exhibited no cytotoxicity against *S. aureus* USA300 and Newman at concentrations ranging from 0 to 160 μM (Fig. 4c and d). Additionally, a paper diffusion experiment was conducted to compare the sizes of the inhibitory zones created by paper disks impregnated with Sch C at 80 μM, OFL at 22 μM, and a combination of both. The results revealed that the size of the inhibitory zone for the combination group was significantly larger than that of the Sch C group (Fig. 4e and f). In comparison to OFL, Sch C exhibited a minimal inhibitory effect on USA300 and Newman *in vitro*. Taken together, these findings demonstrate that Sch C has no direct inhibitory or bactericidal effects on *S. aureus in vitro*. Therefore, we speculated that the observed therapeutic benefits of Sch C in *S. aureus*-induced infections may arise from its antivirulence properties or host-directed mechanisms rather than from classical antimicrobial pathways.

### Sch C directly targeted SrtA, suppressing its activity through stable binding and functional inhibition

**Fig 5:**
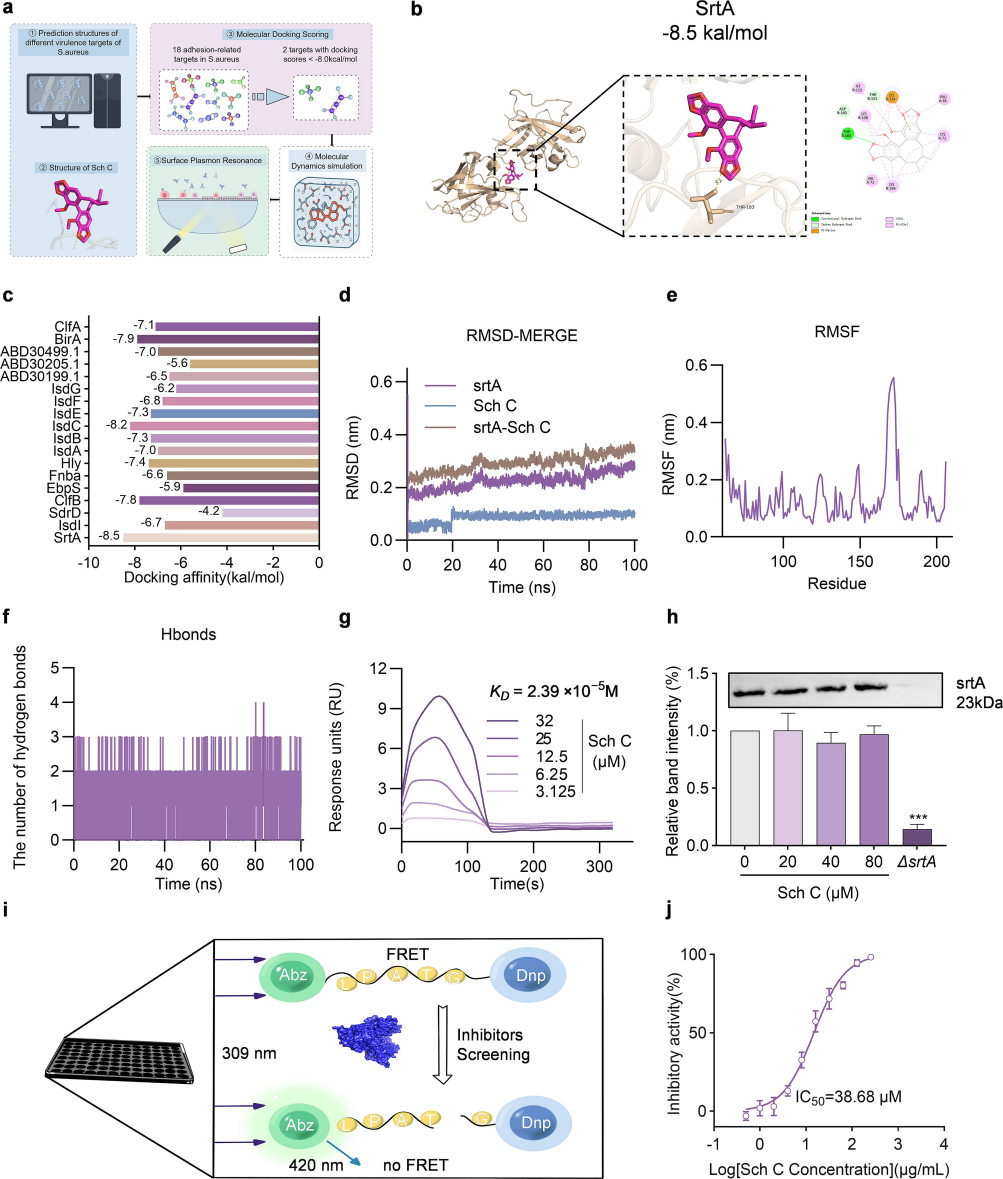
Sch C targets SrtA and suppresses its activity through stable binding and functional inhibition. (**a**) Experimental design for the interaction between Sch C and SrtA (created with Figdraw). (**b**) Molecular docking simulations of Sch C with the above target proteins were performed using AutoDock Vina. The predicted binding conformations with the highest affinity and the corresponding binding free energy values are shown for each protein. (**c**) Binding free energy values of Sch C docked with SrtA, IsdI, SdrD, ClfB, EbpS, FnbA, Hly, IsdA, IsdB, IsdC, IsdE, IsdF, IsdG, ABD30199.1, ABD30205.1, ABD30499.1, BirA, and ClfA proteins. (**d**) Molecular dynamics simulation showing the change in RMSD over time for Sch C, SrtA, and the Sch C-SrtA complex. (**e**) RMSF values of individual amino acid residues in SrtA during molecular dynamics simulation. (**f**) Hydrogen bonding interactions in the Sch C-SrtA complex. (**g**) SPR analysis to determine the binding affinity of Sch C for SrtA. (**h**) Western blot analysis of the effect of different concentrations of Sch C (20, 40, 80, 160 μM) on SrtA protein expression. (**i, j**) Inhibitory effect of Sch C on SrtA activity was evaluated by measuring the fluorescence intensity generated from cleavage of the fluorogenic peptide substrate containing the LPXTG motif. Data are presented as mean ± SD and analyzed using one-way ANOVA: *** indicates *P* < 0.001. All experiments were performed in triplicate.

To elucidate the mechanism by which Sch C alleviates *S. aureus*-induced OM, we integrated molecular docking, dynamic simulations, SPR, Western blotting, and FRET assays to evaluate its inhibitory effect on SrtA (Fig. 5a). Firstly, molecular docking analysis was conducted on key proteins associated with bacterial adhesion. Various virulence proteins related to *S. aureus* adhesion and colonization were selected, including SrtA, IsdI, SdrD, ClfB, EbpS, FnbA, Hly, IsdA, IsdB, IsdC, IsdE, IsdF, IsdG, ABD30199.1, ABD30205.1, ABD30499.1, BirA, and ClfA. The results revealed that among all candidate targets, SrtA exhibited the highest binding affinity with Sch C (docking affinity = −8.5), showing the lowest binding energy and a stable docking conformation (Fig. S4; Fig. 5b and c). The docking simulation results indicated that Sch C stably fit into the active site pocket of SrtA, forming multiple hydrogen bonds and hydrophobic interactions with key amino acid residues such as ASP-160, THR-183, THR-131, and LYS-134. The strength and diversity of these multimodal molecular interactions suggested that Sch C targeted the active site of SrtA with high affinity and specificity, potentially interfering with its biological function (Fig. 5b).

Subsequently, to further investigate the stability of the binding conformation between Sch C and SrtA, molecular dynamics simulations were employed. The results presented in Fig. 5d indicated that the RMSD curve for the Sch C and SrtA complex approached equilibrium after 20 ns, with the average RMSD stabilizing between 0.20 and 0.36 nm. As illustrated in Fig. 5e, residues 70–100, 110–130, 140–160, and 165–180 exhibited increased residue flexibility, with most amino acids fluctuating between 0.0 nm and 0.3 nm. The analysis revealed that the Sch C-SrtA complex formed a maximum of four hydrogen bonds (Fig. 5f). The binding free energy decomposition analysis based on MM/GBSA revealed that individual amino acid residues contributed variably to the total binding energy of the Sch C-SrtA complex (Table S1). SPR analysis showed that Sch C binds to SrtA with a KD of 2.39 × 10⁻⁵ M, indicating moderate affinity (Fig. 5g). Together with the molecular dynamics simulations, these results suggested that Sch C exerts a regulatory effect on SrtA.

Next, to investigate the regulatory role of Sch C on SrtA function, we conducted Western blot to assess the impact of Sch C on SrtA expression after interference with USA300. As illustrated in Fig. 5h, Sch C did not influence the expression of SrtA in *S. aureus*. We employed the FRET method to evaluate whether Sch C acted as an inhibitor of SrtA (Fig. 5i). Further experiments demonstrated that varying concentrations of Sch C significantly inhibited SrtA activity, particularly at higher doses, with an IC_50_ value measured at 38.68 μM (Fig. 5j).

### Sch C alleviated the adhesion and invasion induced by SrtA of *S. aureus*

**Fig 6:**
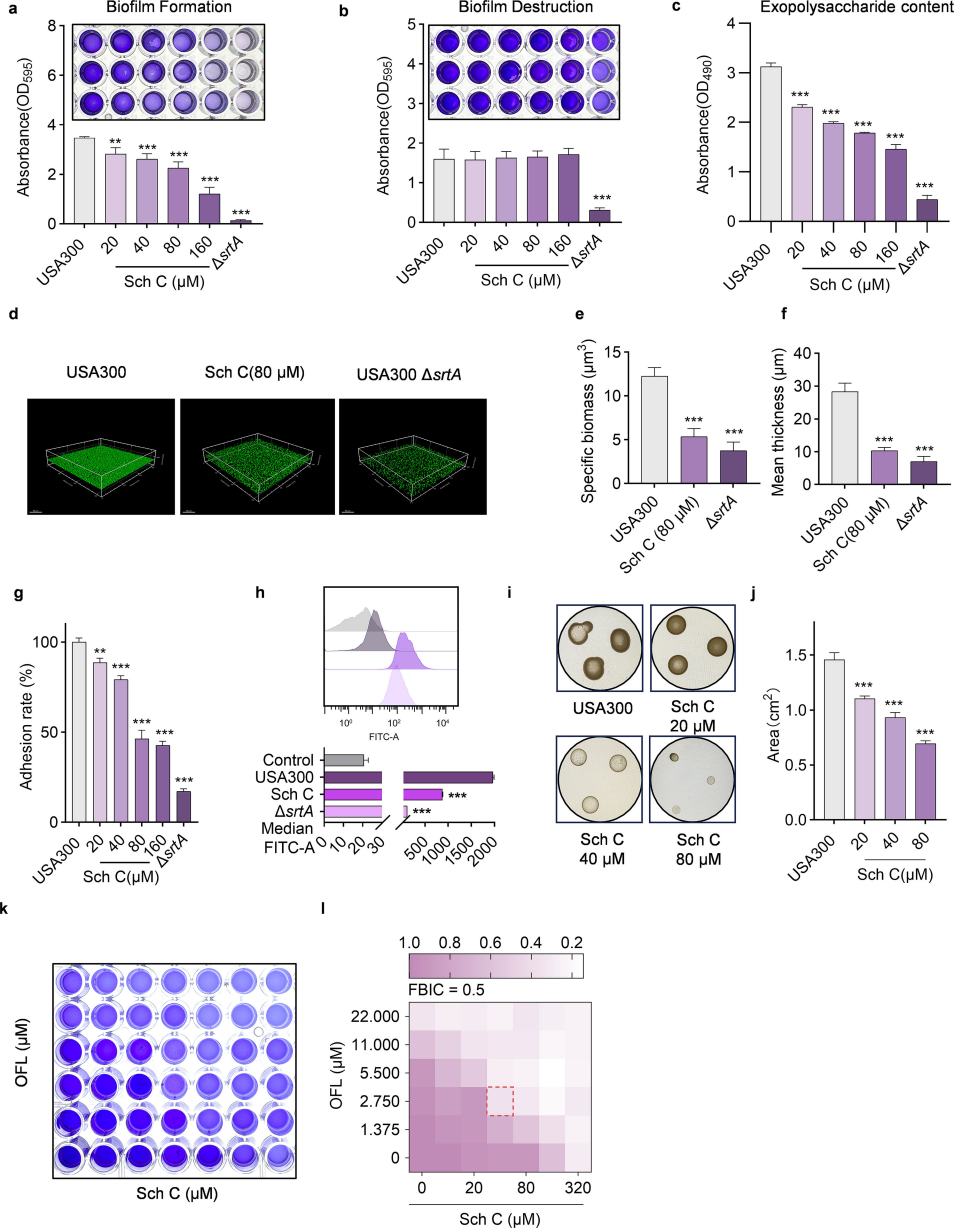
Sch C inhibits SrtA-associated virulence phenotypes in *S. aureus*. (**a, b**) Crystal violet staining was used to assess the effects of Sch C (20 μM–160 μM) on biofilm formation and disruption of preformed biofilms in *S. aureus* USA300. (**c**) Quantification of biofilm-associated polysaccharides revealed a dose-dependent inhibitory effect of Sch C. (**d**) 3D CLSM images of MRSA biofilms stained under different treatments. (**e, f**) Analysis of biofilm by COMSTAT. Measurement of total biofilm biomass and average thickness. (**g**) Adhesion of *S. aureus* USA300 to fibrinogen was evaluated following Sch C treatment. (**h**) Flow cytometry analysis of SpA expression using FITC-conjugated rabbit IgG staining. (**i, j**) Colony spreading area of *S. aureus* USA300 was assessed after treatment with increasing concentrations of Sch C (20, 40, and 80 μM), showing a dose-dependent reduction in movement range. (**k**) Representative crystal violet staining used for the biofilm checkerboard assay. (**l**) The heatmap on the right summarizes the normalized biofilm inhibition pattern across the concentration matrix. Data are expressed as mean ± SD and analyzed by one-way ANOVA: ** indicates *P* < 0.01, *** indicates *P* < 0.001 vs USA300 group.

To evaluate the anti-biofilm properties of Sch C, the following experiments were performed. The crystal violet staining biofilm experiments revealed that Sch C effectively inhibited biofilm formation, particularly at a concentration of 160 μM, leading to a significant reduction in biofilm formation, whereas no significant disruption of preformed biofilms was detected (Fig. 6a and b). Exopolysaccharide content was quantified to evaluate the effect of Sch C on biofilm matrix composition, and treatment with 160 μM Sch C reduced extracellular polysaccharide levels by 36.54% compared with the untreated group (Fig. 6c). CLSM imaging showed that treatment with 80 μM Sch C significantly inhibited USA300 biofilm formation, leading to a 56.35% reduction in biofilm biomass (Fig. 6d and e). In addition, 80 μM Sch C markedly reduced the thickness of *Staphylococcus aureus* biofilms (Fig. 6f).

To investigate whether Sch C inhibited the adhesion of *S. aureus* to host extracellular matrix proteins, *S. aureus* USA300 Δ*srtA* was utilized as a positive control. The results demonstrated a significant reduction in the adhesion ability of the Δ*srtA* group, with an adhesion rate of 17.28 ± 1.33%. In contrast, the adhesion rate for Sch C at 80 μM was 46.21 ± 4.91% (Fig. 6g). These findings suggested that Sch C interferes with fibrinogen binding, thereby affecting the adhesion ability of *S. aureus*. To clarify whether this effect was associated with SrtA-mediated surface protein anchoring, flow cytometry was performed to evaluate SpA presentation on the bacterial surface. The results indicated that, compared to the USA300 group, the fluorescence intensity was lower after treatment with 80 μM Sch C, suggesting that Sch C significantly reduces the levels of SpA displayed on the surface of staphylococci (Fig. 6h). Furthermore, to assess the impact of Sch C on the surface translocation of *S. aureus*, a colony spreading assay was performed. Although *S. aureus* is traditionally considered non-motile due to its lack of flagella, it can expand across semi-solid surfaces via a surfactant-mediated process critical for early biofilm development and tissue colonization (38, 41). The results demonstrated that the colony spreading area of *S. aureus* in the Sch C group was smaller than that in the DMSO group. This suggests that Sch C disrupts the bacterial surface properties necessary for effective spreading (Fig. 6i and j). As shown in Fig. 6k and l, Sch C combined with OFL inhibited USA300 biofilm biomass more effectively than either agent alone. The lowest concentration combination reaching the predefined antibiofilm endpoint gave an FBIC value of 0.5, indicating synergistic antibiofilm activity. Collectively, these findings indicated that Sch C suppressed biofilm formation and bacterial adhesion primarily by disrupting SrtA-dependent anchoring of surface proteins, thereby impairing *S. aureus* biofilm development, adhesion, and immune evasion.

### Safety evaluation of Sch C

To evaluate the *in vivo* toxicity of Sch C, rats were intraperitoneally administered Sch C at concentrations of 20, 40, and 80 μM. Biochemical analysis revealed no significant alterations in liver and kidney function biomarkers, including alanine aminotransferase, aspartate aminotransferase, blood urea nitrogen, and creatinine. Hematological parameters, such as white blood cells and red blood cells, remained within normal ranges across all treatment groups (Fig. S5A through F). Furthermore, histopathological examination of major organs, including the heart, liver, and kidney, by H&E staining revealed no detectable pathological changes (Fig. S5G). These results indicated that Sch C exhibited no apparent systemic toxicity at the tested doses *in vivo*.

## DISCUSSION

MRSA-associated otitis media poses a formidable clinical challenge due to its propensity for biofilm formation, persistent inflammation, and resistance to conventional antibiotics (42). The persistence of biofilms not only compromises the efficacy of standard antibiotic therapy but also drives the emergence of multidrug-resistant strains, complicating the management of OM. In this study, we identified Sch C as a promising therapeutic candidate for MRSA-induced OM. Our findings provide initial evidence that targeting SrtA can attenuate bacterial virulence in OM models, suggesting a potential antivirulence strategy that may serve as a complement to conventional antibiotics. Moreover, this work highlights the dual role of Sch C in both suppressing bacterial pathogenicity and supporting host epithelial barrier integrity, offering a novel therapeutic perspective for managing refractory or resistant OM.

Current therapeutic strategies for OM mainly rely on antibiotics, surgery, and supportive approaches. Antibiotics serve as the first-line treatment for AOM, while systemic and topical agents such as OFL are widely used for CSOM (43, 44). However, their overuse has led to diminished efficacy, particularly in biofilm-associated infections, and has accelerated the emergence of resistant pathogens (45). Although surgical interventions and experimental approaches, including sonodynamic treatment and herbal nanocomposites, have been explored, they offer only partial solutions and do not adequately address the challenges of chronicity and resistance (45, 46). To date, the concept of antivirulence therapy has been investigated primarily in pneumonia and systemic infections but has rarely been applied to otitis media (47). Our study provides the preclinical evidence that Sch C exerts therapeutic effects in MRSA-induced OM by selectively targeting SrtA, thereby attenuating bacterial virulence. These findings support the feasibility of an antivirulence strategy as a complementary approach to current OM treatment.

*Schisandra chinensis* has been extensively investigated for its anti-inflammatory, antioxidant, and hepatoprotective effects, with lignans recognized as its major bioactive constituents (48). Previous studies demonstrated that schizandrin suppresses inflammatory mediators such as TNF-α, IL-6, IL-1β, NO, and PGE2, thereby exerting protective effects in models of pneumonia, osteoarthritis, and acute lung injury (49–51). In parallel, aqueous extracts of *Schisandra chinensis* have been reported to display antibacterial activity, particularly against *Staphylococcus aureus*, although the active constituents and precise mechanisms remain undefined (52). To date, no reports have described the direct antibacterial activity of Sch C or its efficacy in alleviating OM. Our findings suggest that Sch C attenuates *S. aureus* virulence through a mechanism associated with SrtA inhibition, indicating a mode of action distinct from conventional bactericidal therapy. This expands the known pharmacological profile of Sch C and supports the relevance of SrtA-directed antivirulence intervention in refractory ear infections.

Virulence factors such as toxins, adhesins, and immune evasion proteins enable pathogens to damage host tissues and escape immune clearance. Among these determinants, SrtA has emerged as a particularly attractive antivirulence target, as it anchors LPXTG motif-containing proteins that are indispensable for adhesion, immune evasion, and biofilm development (53). Moreover, SrtA-dependent anchoring of surface proteins is indispensable for biofilm formation, where it promotes initial adhesion, supports matrix accumulation, and enhances bacterial tolerance to antibiotics and host immune clearance (54). Notably, deletion of *srtA* profoundly attenuates *S. aureus* virulence in multiple animal models without affecting viability (55, 56). Consistently, our study revealed that Sch C reduced the surface exposure of adhesins such as SpA, diminished fibrinogen binding, suppressed exopolysaccharide production, and ultimately impaired early biofilm formation. In the HMEECs infection model, Sch C significantly decreased LDH release and increased the Calcein/PI viability ratio, phenocopying the effects observed in the Δ*srtA* mutant and further supporting an SrtA-mediated protective mechanism. Importantly, Western blot analysis confirmed that Sch C does not affect SrtA expression, suggesting that it interferes directly with its enzymatic activity. Molecular docking further revealed that among 18 adhesion-related virulence proteins, SrtA exhibited the lowest binding energy, indicating significant inhibitory potential. Collectively, these findings demonstrate that Sch C directly targets SrtA, providing strong mechanistic support for its use as an antivirulence agent. Notably, the *in vitro* assays were designed to define the molecular basis of Sch C action rather than to recapitulate the full pathological spectrum of OM.

The *in vivo* data further extend these observations in distinct disease contexts. Sch C exhibited pronounced therapeutic efficacy in both AOM and CSOM models. In the AOM model, Sch C alleviated tympanic membrane opacity, mucosal thickening, and inflammatory infiltration, while significantly suppressing NF-κB activation and reducing pro-inflammatory cytokines. By contrast, the CSOM model reflects the persistent and biofilm-associated stage of disease. In this model, Sch C restored TM integrity, reduced epithelial thickening, suppressed MUC5B overexpression, and reversed MRSA-induced downregulation of ZO-1, suggesting a capacity to repair epithelial barrier disruption. This is particularly relevant as epithelial barrier dysfunction is increasingly recognized as a critical driver of chronic otitis pathology, paralleling mechanisms described in intestinal and airway inflammatory diseases (22, 57, 58). Moreover, given that biofilm formation underlies the chronicity and antibiotic tolerance of CSOM, improvements in epithelial integrity may also be associated with disruption of SrtA-dependent surface proteins such as SpA (59). Taken together, the in *vitro*, AOM, and CSOM models address different but complementary aspects of MRSA-induced otitis media, namely molecular mechanisms, early colonization, acute injury, and persistent infection with chronic epithelial damage, respectively. This integrated evidence supports the conclusion that Sch C exerts a dual protective effect by attenuating bacterial virulence while preserving host epithelial barrier integrity.

The remarkable synergistic effect observed in the combination group (Sch C + OFL) warrants further mechanistic consideration. Based on the current evidence, this interaction is more likely attributable to Sch C-mediated potentiation of OFL activity rather than the reverse. Although USA300 is an MRSA strain, methicillin resistance does not necessarily imply complete resistance to ofloxacin, and OFL retained measurable *in vitro* activity against the study strain. In this context, the enhanced efficacy of the combination may be explained, at least in part, by attenuation of virulence-related processes, reduced bacterial adherence, and impaired early biofilm establishment. Unlike conventional strategies that rely primarily on bactericidal pressure, this therapeutic pattern may involve functional disarming of the pathogen rather than direct elimination alone. By inhibiting SrtA, Sch C may interfere with the anchoring of surface adhesins and other virulence-associated proteins, thereby weakening adherence- and biofilm-related persistence and improving the accessibility and effectiveness of OFL under infection-related conditions. This interpretation is also consistent with our observations that Sch C alone showed limited direct antibacterial activity, whereas the Sch C-OFL combination displayed synergistic antibiofilm activity *in vitro*. Accordingly, the combination treatment was associated with greater improvement in tissue pathology and inflammatory profiles than either intervention alone. Nevertheless, the precise molecular directionality of this interaction remains to be further validated.

An important implication of this study is that Sch C may act at the host-pathogen interface through complementary mechanisms. Our data suggest that its therapeutic effect likely derives from the combined attenuation of bacterial virulence and mitigation of host inflammatory injury. Structurally, the lipophilic dibenzocyclooctadiene scaffold allows Sch C to navigate both the bacterial surface and the host intracellular environment. By interacting with SrtA and reducing surface adhesin anchoring, Sch C may lessen the initial pathogenic burden on the middle ear mucosa. At the same time, suppression of NF-κB-associated inflammatory signaling may help limit excessive inflammation, epithelial barrier disruption, and mucus hypersecretion. Thus, Sch C alleviates MRSA-induced OM through a synchronized mechanism that suppresses pathogen pathogenicity and restores host epithelial function, effectively breaking the vicious cycle of chronic inflammation and tissue remodeling.

While our findings establish Sch C as a potent antivirulence agent against MRSA-induced OM, direct *in vivo* visualization of bacteria or biofilm was not performed. Nevertheless, the attenuation of chronic tissue remodeling and consistency with *in vitro* assays—including Δ*srtA* mutant verification—support Sch C’s potential to interfere with biofilm-mediated persistence. Pre-experiments indicated that knockout strains often fail to establish stable infections due to reduced virulence and impaired colonization, limiting their use for *in vivo* validation, so wild-type USA300 was used to assess treatment efficacy in a clinically relevant model. Future studies should address pharmacokinetics, safety, long-term toxicity, structure-activity optimization, improved local delivery, and rational combination with conventional antibiotics to enhance therapeutic potential.

**Fig 7:**
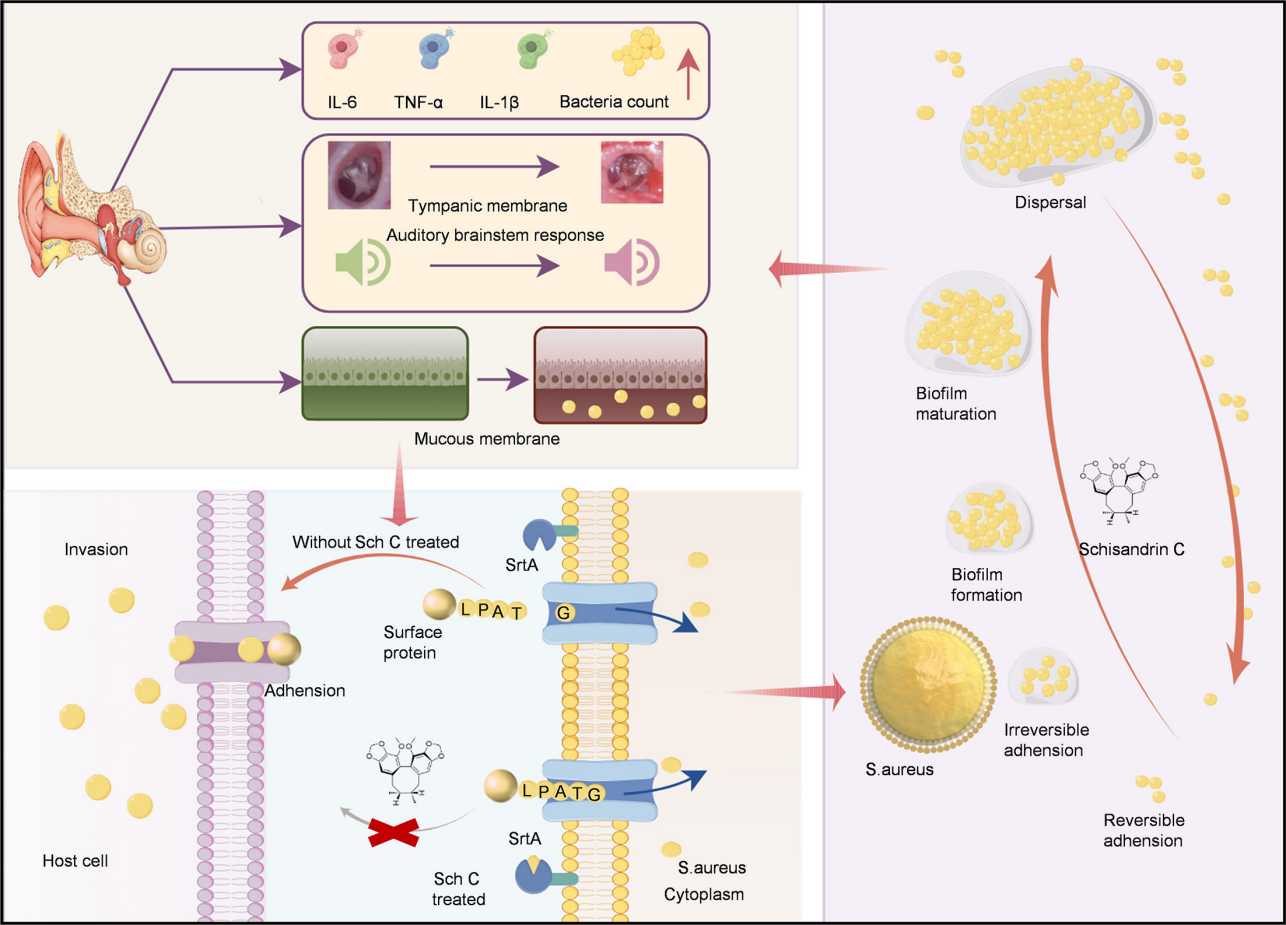
Sch C inhibits the virulence of *S. aureus* by targeting SrtA. Sch C inhibits the virulence of *S. aureus* by targeting SrtA, a key enzyme involved in bacterial pathogenesis. SrtA plays a critical role in anchoring surface proteins to the bacterial cell wall, facilitating bacterial adhesion to host cells, invasion, and biofilm formation. By inhibiting SrtA, Sch C effectively disrupts these crucial steps, reducing *S. aureus* colonization and spreading in host tissues, thereby diminishing its pathogenicity. Furthermore, in *vivo* models of AOM and CSOM, Sch C demonstrated significant therapeutic effects by alleviating inflammation and promoting tissue repair. These findings suggest that Sch C, through targeting SrtA, provides a promising therapeutic approach for treating *S. aureus*-mediated infections.

In summary, this study elucidates a novel dual anti-infective mechanism of Sch C. It directly targets bacterial virulence by inhibiting SrtA to impede adhesion, and concurrently fortifies host defense by restoring epithelial barrier function (Fig. 7a). These findings position Sch C as a promising natural-product-derived therapeutic candidate for recalcitrant MRSA-associated OM, thereby informing novel strategic directions for combating antibiotic resistance.

## AFTER EPUB

*[This article was published on 10 June 2026 with an error in the Fig. 7 caption. The caption was corrected in the current version, posted on 15 June 2026.]*

## SUPPLEMENTAL MATERIAL

Supplemental material aac.00095-26-s0001.docx Fig. S1 to S5; Table S1.

ASM does not own the copyrights to Supplemental Material that may be linked to, or accessed through, an article. The authors have granted ASM a non-exclusive, world-wide license to publish the Supplemental Material files. Please contact the corresponding author directly for reuse.
