# Supplementary material for: The protective effect of Schisandrin C against methicillin-resistant Staphylococcus aureus-induced otitis media
Source: Antimicrob Agents Chemother. 2026 Jun 10;70(7):e00095-26. doi: 10.1128/aac.00095-26 (PMC13321823; doi:10.1128/aac.00095-26)
Supplement: Supplemental material — Fig. S1 to S5; Table S1. [file aac.00095-26-s0001.docx]

**Supplementary Information**

**The Protective Effect of Schisandrin C against Methicillin-Resistant Staphylococcus aureus - Induced Otitis Media**

Weifang Sun^a^, Meihui Tian^b^, Xingye Wang^a^, Shuang Jiang^c^, Mengli Jin^d^, Yan Wang^e^, Yating Tang^a^, Shuyue Zhu^f^, Wenlu Liao^e^, Xueying Ding^e^, Xuanyu Lv^e^, Huan Liu^a^, Wu Song^d*^, Lin Wei^d*^, Yong Tang ^eg*^

*^a^* *Traditional Chinese Medicine College, Changchun University of Chinese Medicine, Changchun 130117, Jilin, China.*

*^b^* *Department of Traditional Chinese Medicine, the Fourth Affiliated Hospital of School of Medicine, and International School of Medicine, International Institutes of Medicine, Zhejiang University, 322000, Zhejiang, China.*

*^c^ School of Health Management, Changchun University of Chinese Medicine, Changchun 130117, China*

*^d^* *School of Basic Medical Science, Changchun University of Chinese Medicine, Changchun 130117, China*

*^e^* *College of Integrated Chinese and Western Medicine, Changchun University of Chinese Medicine, Changchun 130117, China*

*^f^* *School of Pharmaceutical Sciences, Changchun University of Chinese Medicine, Changchun 130117, China*

*^g^* *Department of Otorhinolaryngology – Head and Neck Surgery, The People’s Hospital of Jilin Province, Changchun, 130021, China*

*Correspondence authors:

Wu Song, [five841110@126.com](mailto:five841110@126.com), Changchun University of Chinese Medicine, Changchun 130117, Jilin, China.

Lin Wei, lynnw2013@sina.com, Changchun University of Chinese Medicine, Changchun 130117, Jilin, China.

Yong Tang, tangyong@ccucm.edu.cn, Changchun University of Chinese Medicine, Changchun 130117, Jilin, China. Department of Otorhinolaryngology – Head and Neck Surgery, The People’s Hospital of Jilin Province, Changchun, 130021, China.

**Supplementary Figure.1** Quality test report of Schisandrin C (Sch C).

**Supplementary Figure.2** Animal experiment-related tissue dissection and typical pathological manifestations.

**Supplementary Figure.3** SDS-PAGE grayscale images of purified proteins sortase A (SrtA).

**Supplementary Figure.4** Molecular docking results of Sch C with adhesion-related targets in *Staphylococcus aureus (S. aureus)*.

**Supplementary Figure.5** Safety Evaluation of Sch C.

**Supplementary Table.1** The energy contributions of individual amino acids with individual energy components contribute to the total MMGBSA binding energy for the Sch C - SrtA complex, including Δ *Evdw*, Δ *Eele*, Δ *EGB*, Δ *Esurf* and Δ *Etotal.*

**Supplementary Figure.1** Quality test report of Sch C.


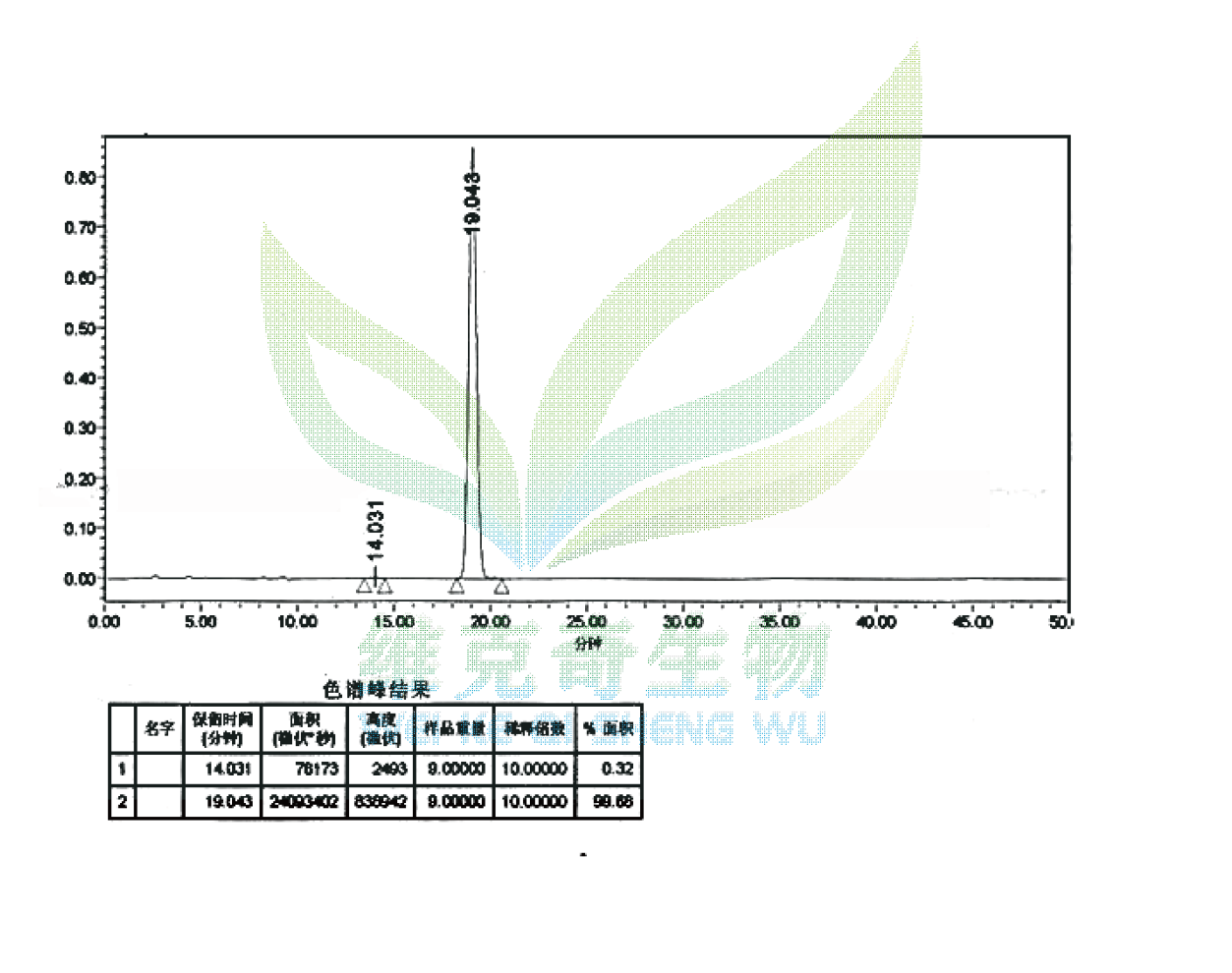


|  | Name | Retention Time (minutes) | Peak Area (mV·s) | Height (mV) | Sample Volume (μL) | Standard Concentration (%) | Purity |
| --- | --- | --- | --- | --- | --- | --- | --- |
| 1 |  | 14.031 | 76173 | 2493 | 9.00000 | 10.00000 | 0.32 |
| 2 |  | 19.043 | 2408343 | 838842 | 9.00000 | 10.00000 | 99.68 |

Figure S1. High Performance Liquid Chromatography (HPLC) chromatogram of Sch C. The purity of Sch C was 99.68%.

**Supplementary Figure.2** Animal experiment-related tissue dissection and typical pathological manifestations.

**A B C D**


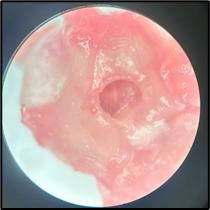

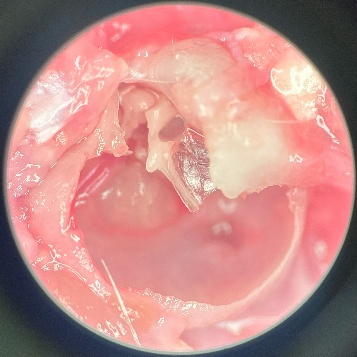

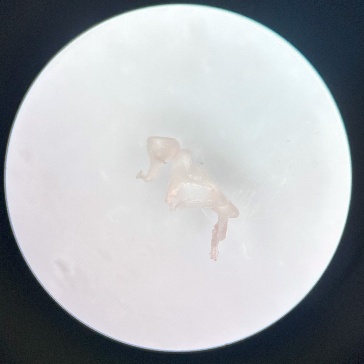


Malleus

Incus

Stapes


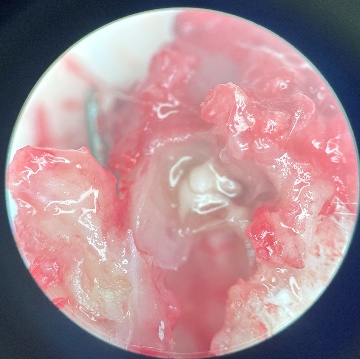


**E**


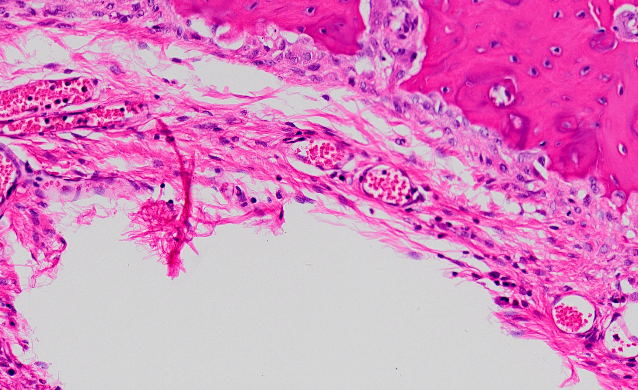


50 μm

Figure S2. (A) Distant view of normal rat tympanic bulla and tympanic membrane under microscopy. (B) Normal middle ear mucosa appears thin, smooth, and free of effusion. (C) Rat auditory ossicles. (D) Rice-water-like purulent exudate observed 1-3 days after AOM modeling. (E) CSOM: HE staining reveals abundant fibroblasts in the deep mucosal layer.

**Supplementary Figure.3** SDS-PAGE grayscale images of purified proteins SrtA.

**Marker**

**1**

**2**

**3**

**4**

**5**

**6**

**7**

**8**


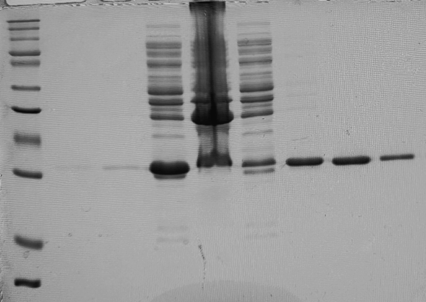


**200—**

**kDa**

**90—**

**110—**

**140—**

**68—**

**53—**

**40—**

**SrtA**

**23kDa**

**30—**

**—**

**20—**

**13—**

**8—**

Figure S3. The full SDS-PAGE grayscale images of purified proteins SrtA. Lanes 1 and 2 illustrate the expression of the BL21 (DE3) pET28a::SrtA protein preinduction and postinduction with IPTG, respectively. Lanes 3 and 4 represent the bacterial supernatant and pellet obtained post ultrasonic disruption and centrifugation, respectively. Lane 5 indicates nonspecific proteins eluted at a low imidazole concentration of 10 mM. The proteins obtained at a moderate imidazole concentration of 50 mM are displayed in lane 6, while lanes 7 and 8 exhibit the target SrtA protein, which was purified under stringent conditions using 100 mM and 200 mM imidazole, respectively.

**Supplementary Figure.4** Molecular docking results of Sch C with adhesion-related targets in *S. aureus*.


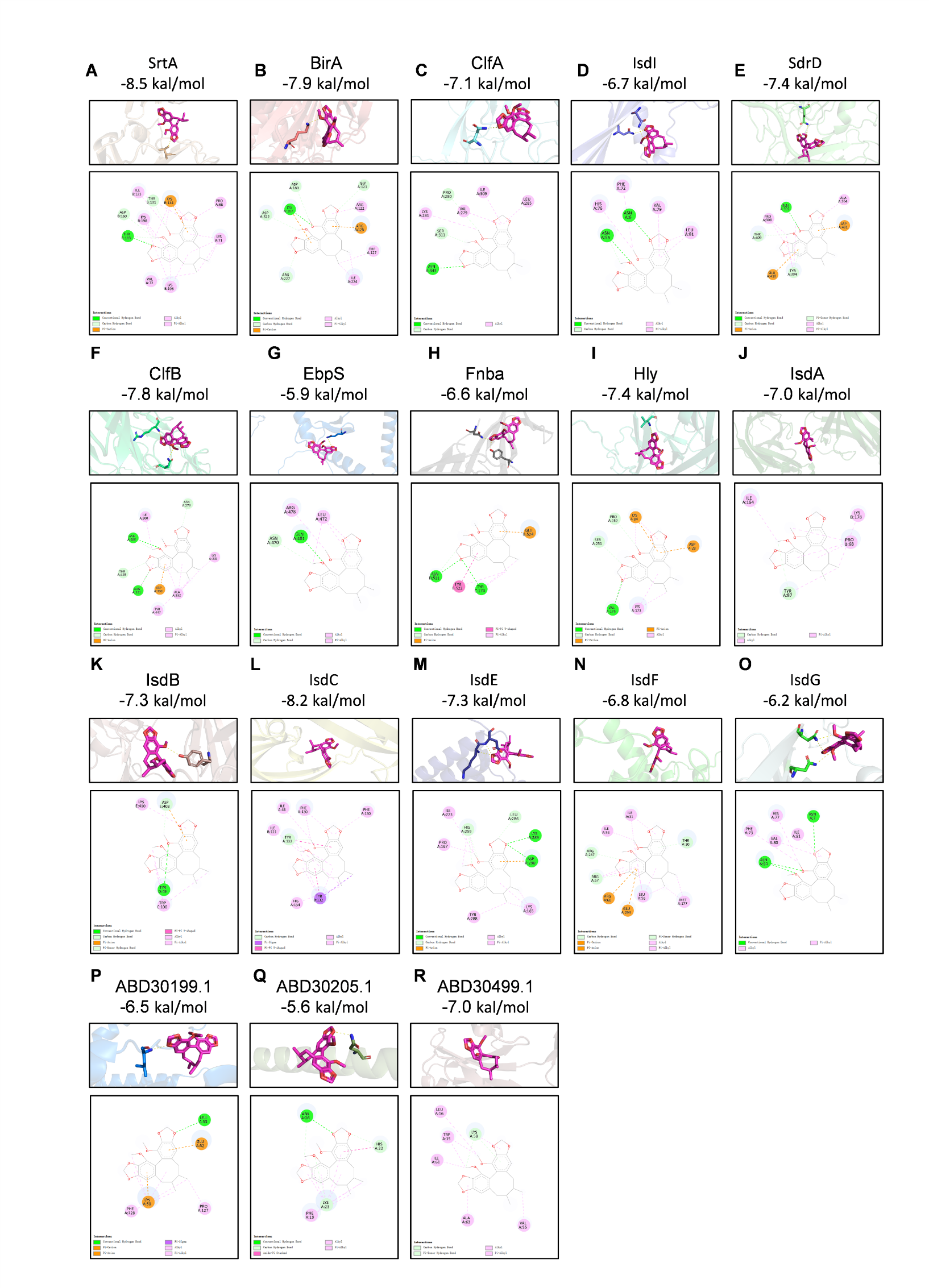


Figure S4. (A-R) Molecular docking models. Microscopic 3D and 2D molecular docking models of SrtA-Sch C with multiple targets, including BirA, ClfA, IsdA, IsdB, IsdC, IsdE, IsdF, IsdG, IsdL, SdrD, ClfB, EbpS, Fnba, Hly, ABD30199.1, and ABD30499.1.

**Supplementary Figure.5** Safety Evaluation of Sch C.


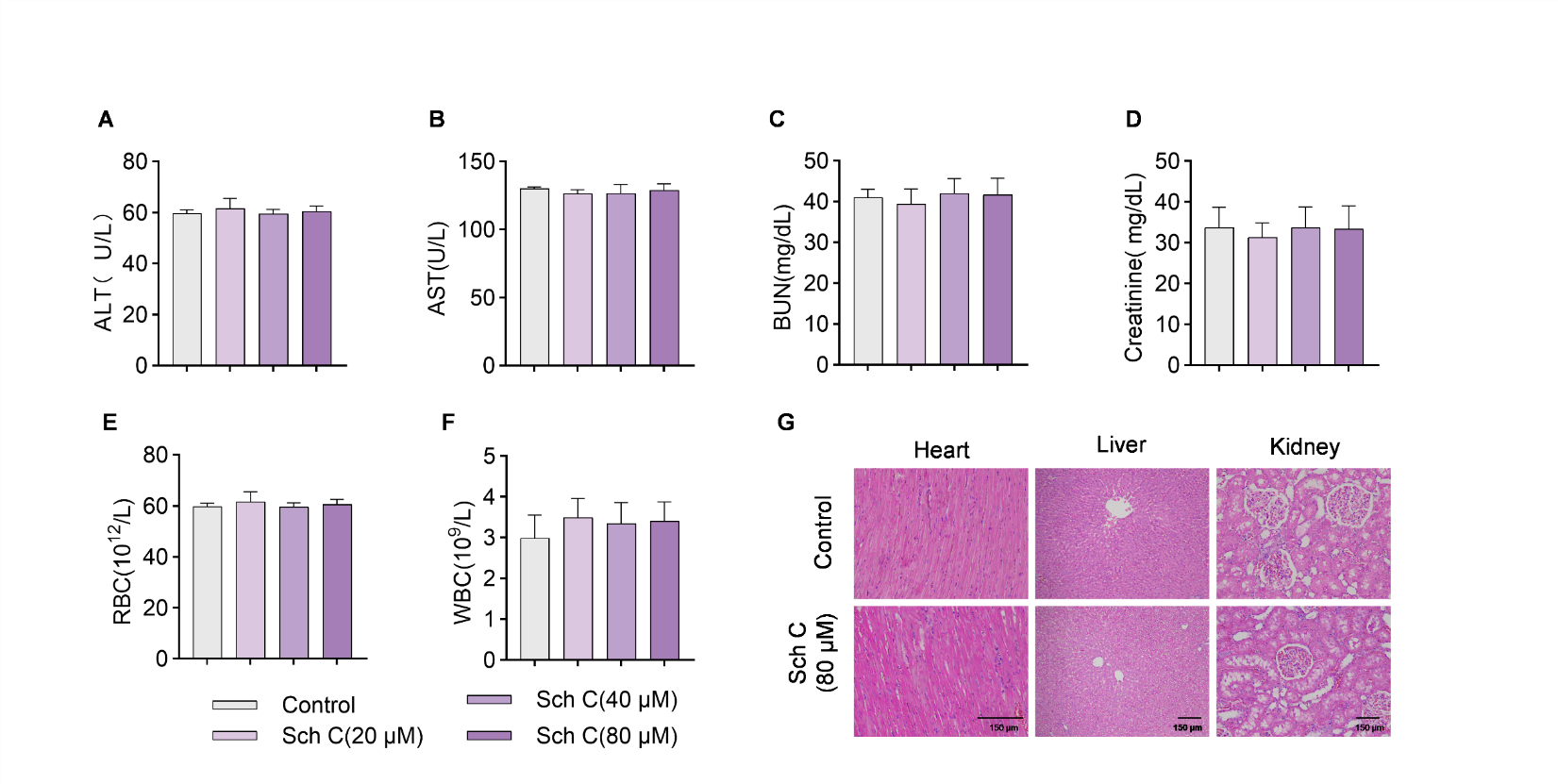


Figure S5. (A–F) Blood biochemical and hematological parameters of the liver and kidneys were assessed. (G) Histological evaluation for toxicological analysis was performed using H&E staining on paraffin-embedded sections of major organs from rats, revealing minimal to no pathological alterations. Abbreviations: ALT, alanine aminotransferase, AST, aspartate aminotransferase, BUN, blood urea nitrogen, CREA, creatinine, RBC, red blood cells, WBC, white blood cells.

**Supplementary Table.1** The energy contributions of individual amino acids with individual energy components contribute to the total MMGBSA binding energy for the *Sch C* -*SrtA* complex, including Δ *Evdw*, Δ *Eele*, Δ *EGB*, Δ *Esurf* and Δ *Etotal.*

**A**

|  | Δ *Evdw** | Δ *Eele** | Δ *EGB** | Δ *Esurf* | * | Δ *Etotal* | * |
| --- | --- | --- | --- | --- | --- | --- | --- |
| R:A:GLN:113 | -0.274 | 0.058 | -0.347 | -0.000 | | -0.563 | |
| R:A:LYS:138 | -0.889 | -0.465 | 1.450 | -0.133 | | -0.037 | |
| R:A:LYS:154 | -0.984 | -0.178 | 3.871 | -0.224 | | 2.484 | |
| R:A:MET:155 | -0.349 | -0.365 | 0.111 | 0.000 | | -0.604 | |
| R:A:THR:156 | -2.632 | -1.115 | 1.408 | -0.195 | | -2.535 | |
| R:A:SER:157 | -1.305 | -0.050 | 0.902 | -0.175 | | -0.628 | |
| R:A:ILE:158 | -0.513 | 0.043 | 0.023 | -0.065 | | -0.512 | |
| R:A:ASP:170 | -1.362 | -0.228 | 1.698 | -0.157 | | -0.049 | |
| R:A:LYS:173 | -2.919 | -1.504 | 4.206 | -0.495 | | -0.711 | |
| R:A:LYS:175 | -0.178 | 0.874 | -0.539 | -0.003 | | 0.153 | |
| R:A:ASP:176 | -0.702 | -1.141 | 1.996 | -0.152 | | 0.001 | |
| R:A:LYS:177  R:A:GLN:178  R:A:VAL:201  R:A:THR:203 | -0.204  -1.239  -0.825  -1.879 | 0.084  0.301  0.086  -2.608 | -0.008  0.581  -0.181  2.056 | -0.000  -0.045  -0.044  -0.318 | | -0.129  -0.403  -0.964  -2.749 | |


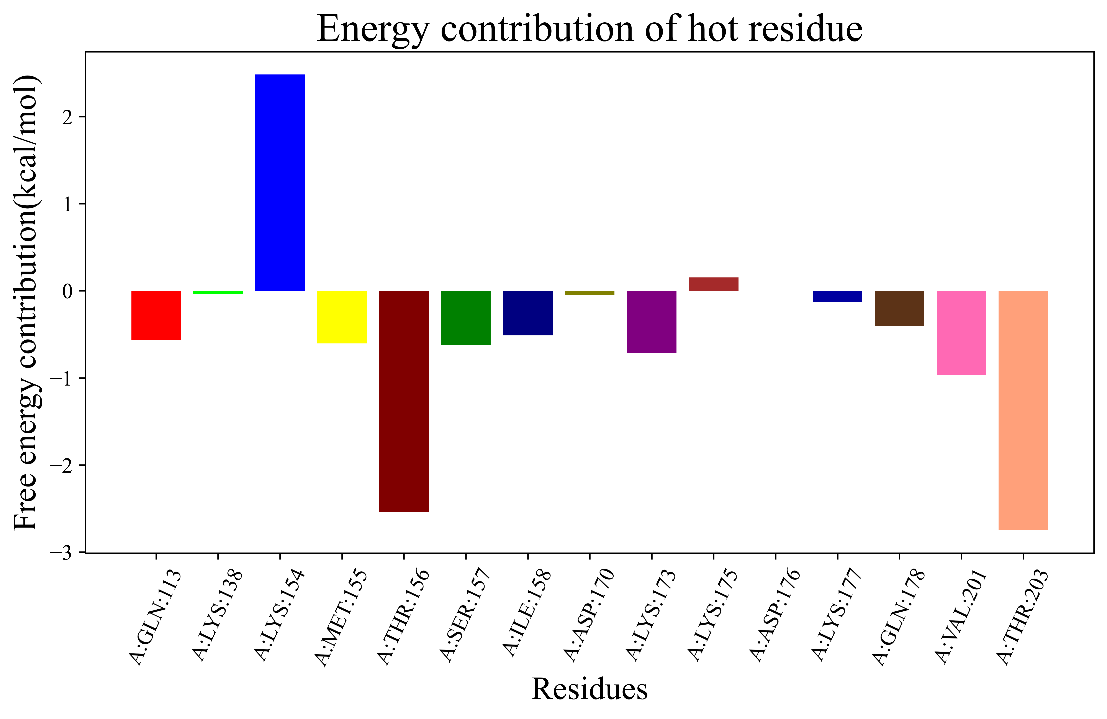
*Δ *Evdw*, Δ *Eele*, Δ *EGB* and Δ *Esurf* and Δ *Etotal* represent van der Waals, electrostatics, polar solvation, nonpolar solvation and total MMGBSA binding energy, respectively.

**B**

| Energy | Schisandrin C-SrtA complex (kcal/mol) |
| --- | --- |
| Δ *Evdw* | -36.32 |
| Δ *Eele* | -11.13 |
| Δ *EGB* | 26.39 |
| Δ *Esurf* | -4.66 |
| Δ *Etotal* | -25.72 |

Table. S1. (A)The energy contributions of individual amino acids. (B) Contribution of energy components to the total MMGBSA binding energy of the Sch C-SrtA complex. The total binding free energy (-25.72 kcal/mol) is negative, confirming that the binding of Sch C to SrtA is thermodynamically spontaneous and primarily driven by the synergistic effects of hydrophobic interactions and electrostatic forces.
